# SREBP2 restricts osteoclast differentiation and activity by regulating IRF7 and limits inflammatory bone erosion

**DOI:** 10.1038/s41413-024-00354-4

**Published:** 2024-08-27

**Authors:** Haemin Kim, In Ah Choi, Akio Umemoto, Seyeon Bae, Kaichi Kaneko, Masataka Mizuno, Eugenia Giannopoulou, Tania Pannellini, Liang Deng, Kyung-Hyun Park-Min

**Affiliations:** 1 Arthritis and Tissue Degeneration Program, David Z. Rosensweig Genomics Research Center, Hospital for Special Surgery, New York, NY 11366 USA; 2 Department of Medicine, Weill Cornell Medical College, New York, NY 10021 USA; 3 CHA Biomedical Research Institute, CHA Bundang Medical Center, CHA University School of Medicine, Seongnam, 13496 Republic of Korea; 4 Department of Internal Medicine, College of Medicine, Chungbuk National University, Cheongju, Chungbuk 28644 Republic of Korea; 5 Biological Sciences Department, New York City College of Technology, City University of New York, Brooklyn, NY 11201 USA; 6 Dermatology Service, Department of Medicine, Memorial Sloan Kettering Cancer Center, New York, NY 10065 USA; 7 Department of Dermatology, Weill Cornell Medical College, New York, NY 10021 USA; 8 BCMB Allied Program, Weill Cornell Graduate School of Medical Sciences, New York, NY 10065 USA

**Keywords:** Bone, Physiology

## Abstract

Osteoclasts are multinucleated bone-resorbing cells, and their formation is tightly regulated to prevent excessive bone loss. However, the mechanisms by which osteoclast formation is restricted remain incompletely determined. Here, we found that sterol regulatory element binding protein 2 (SREBP2) functions as a negative regulator of osteoclast formation and inflammatory bone loss. Cholesterols and SREBP2, a key transcription factor for cholesterol biosynthesis, increased in the late phase of osteoclastogenesis. The ablation of SREBP2 in myeloid cells resulted in increased in vivo and in vitro osteoclastogenesis, leading to low bone mass. Moreover, deletion of SREBP2 accelerated inflammatory bone destruction in murine inflammatory osteolysis and arthritis models. SREBP2-mediated regulation of osteoclastogenesis is independent of its canonical function in cholesterol biosynthesis but is mediated, in part, by its downstream target, interferon regulatory factor 7 (IRF7). Taken together, our study highlights a previously undescribed role of the SREBP2-IRF7 regulatory circuit as a negative feedback loop in osteoclast differentiation and represents a novel mechanism to restrain pathological bone destruction.

## Introduction

Osteoclasts are myeloid-derived sole bone-resorbing cells that play essential roles in bone remodeling, and their dysregulation leads to pathological bone loss, such as osteoporosis and bone erosion in patients with rheumatoid arthritis (RA). Therefore, osteoclastic activities are tightly regulated in physiologic states by the balance between positive feedforward and negative feedback signals to prevent excessive bone loss and minimize bone damage.^1,2^ While positive regulatory signal mechanisms mediated by receptor activator of nuclear factor-κB ligand (RANKL) and inflammatory molecules, including tumor necrosis factor-α (TNFα), are extensively studied,^3^ relatively little is known about the mechanisms of the negative regulators in osteoclasts. Negative regulatory signals in osteoclastogenesis known so far include osteoprotegerin (OPG, TNFRSF11B), leucine-rich repeat-containing G-protein-coupled receptor 4, cytokines such as interferons (IFNs) and interleukin-10, and transcription factors such as B-cell lymphoma 6, interferon regulatory factor-8 (IRF8), MafB, inhibitors of differentiation/DNA binding-2, and Eos.^2,4–10^ Given the importance of interlinking positive and negative feedback mechanisms, a better understanding of the negative feedback mechanisms during osteoclastogenesis would provide insights into potential targets to limit excessive osteoclast activity in pathological states, such as inflammatory arthritis. However, studies on negative regulators focus mainly on the early phase of osteoclastogenesis.

Sterol regulatory element binding protein-2 (SREBP2) is a crucial transcription factor that controls the cholesterol biosynthesis pathway.^11^ and inflammasome activation.^12^ Our previous study also showed that SREBP2 can be activated by TNF, and SREBP2 and its target gene expressions are elevated in synovial macrophages from patients with RA, implicating the role of SREBP2 in the context of chronic TNF-driven inflammatory conditions.^13^ SREBP cleavage activating protein (SCAP) is a sterol-regulated protein that facilitates proteolytic cleavage-mediated activation of SREBPs.^14^ Previous studies have established that inhibiting the SREBPs/SCAP system suppressed osteoclast differentiation and activity, suggesting a positive role of SREBP2 in osteoclastogenesis.^15–17^ Fatostatin, which blocks the translocation of SREBPs to Golgi apparatus, inhibited RANKL-induced osteoclastogenesis and bone loss.^15,16^ Anhydroicaritin, a natural product to inhibit SREBPs, suppressed osteoclastogenesis.^17^ However, those inhibitors can affect all the isoforms of SREBPs (SREBP1a, SREBP1c, and SREBP2), and the effect of SREBP2 in vivo has not been directly characterized.

In this study, we identify that SREBP2 is a negative regulator in the late phase of osteoclastogenesis. Notably, myeloid-specific SREBP2 deficient mice exhibit reduced trabecular bone mass compared with control mice, and ablation of SREBP2 in myeloid cells resulted in increased in vivo and in vitro osteoclastogenesis. Moreover, SREBP2 deficiency exacerbates TNFα-induced osteolysis and accelerates inflammatory bone destruction in mice, supporting a protective role of SREBP2 in inflammatory bone destruction. Mechanistically, SREBP2 transcriptionally induced IRF7 and suppressed osteoclastogenesis via IRF7, highlighting that the SREBP2/IRF7 axis serves as a novel negative regulator at the late stage of osteoclastogenesis. Taken together, these findings identify a novel function and mechanism by which SREBP2 suppresses osteoclast-mediated bone loss and provides insights into a negative feedback mechanism for osteoclast-mediated bone loss, which is crucial to develop new therapeutic strategies to combat inflammatory bone destruction.

## Results

### Myeloid-specific deletion of SREBP2 causes decreased bone mass and increased osteoclastogenesis under physiological conditions

**Fig. 1:**
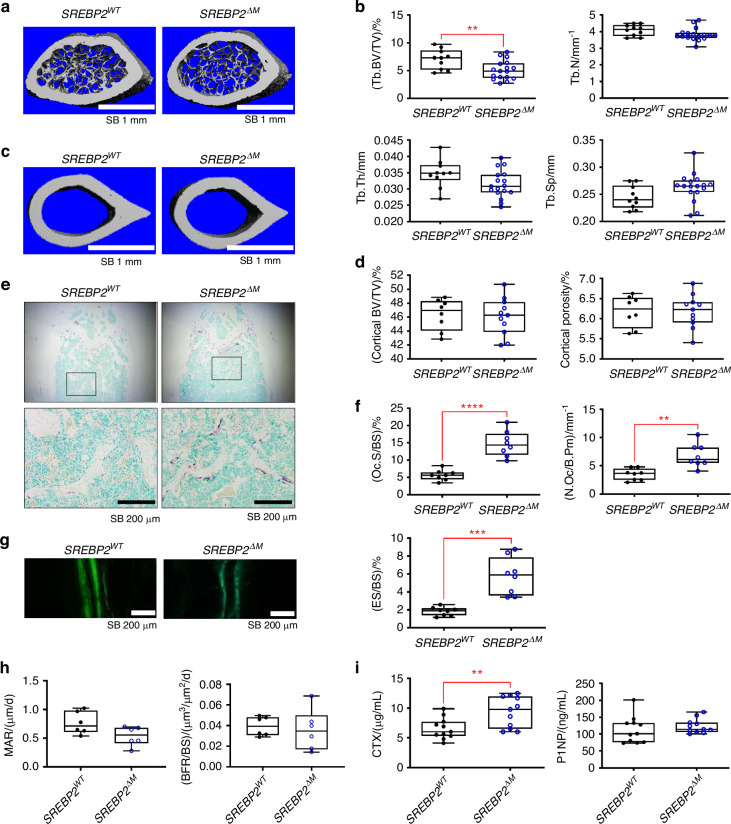
SREBP2 protects mice from bone loss. **a**–**d** Femurs from 12-week-old female *SREBP2*^*WT*^ and littermate control *SREBP2*^*∆M*^ mice were subjected to microCT scan analysis. Representative 3D images of trabecular bone (**a**) or cortical bone (**c**) of distal femurs were shown. Scale bars: 1 mm. **b** Trabecular bone parameters of femurs from *SREBP2*^*WT*^ (*n* = 10) and *SREBP2*^*∆M*^ (*n* = 17) mice. Bone volume/ tissue volume (Tb. BV/TV), trabecular space (Tb. Sp), trabecular thickness (Tb.Th), and trabecular numbers (Tb.N). **d** Cortical bone parameters of *SREBP2*^*WT*^ (*n* = 8) and *SREBP2*^*∆M*^ (*n* = 11) mice. Bone volume per tissue volume (BV/TV) and porosity. **e**, **f** Histological assessment of femurs from 12-week-old female *SREBP2* ^*WT*^ and *SREBP2*^*∆M*^ mice (*n* = 8 per group). **e** Representative images. Decalcified sections of distal femurs of *SREBP2* ^*WT*^ and *SREBP2*^*∆M*^ mice were shown after TRAP staining. Methyl Green staining was followed to indicate bone. Scale bars: 200 µm. **f** The osteoclast surface per bone surface (Oc. S/BS), the number of osteoclasts per bone perimeter (N. Oc/B.Pm), eroded surface per bone surface (ES/BS) were quantified. **g**, **h** Bone dynamic labeling analysis from 12-week-old female *SREBP2* ^*WT*^ and *SREBP2*^*∆M*^ mice. **g** Representative images of calcein labeling. Scale bars: 200 µm. **h** Mineral apposition rate (MAR) and bone formation rate per bone surface (BFR/BS) (*n* = 6 per group). **i** The measurement of C-telopeptide of collagen type 1 (CTX) and Procollagen type I N-terminal propeptide (P1NP) from 12-week-old female *SREBP2* ^*WT*^ and *SREBP2*^*∆M*^ mice (*n* = 10 per group). All data are shown as median and interquartile range. n.s., not significant; **P < 0.01, ***P < 0.001, ****P < 0.000 1 by two-tailed unpaired *t* test

To examine the role of SREBP2 in osteoclastogenesis in vivo, we generated myeloid cell-specific SREPB2 deficient mice by crossing LysM-Cre mice with *Srebp2*^*fl/fl*^ mice (*Srebp2*^*fl/fl*^ *LysM-Cre*, referred to as *SREBP2*^*ΔM*^ mice), and compared their bone mass with age- and sex-matched littermate *SREBP2*^*+/+*^ *LysM-Cre* mice (control, referred to as *SREBP2*^*WT*^ mice). The body weight and femur length were also unaffected by SREBP2 deletion (Fig. S1a, b). Microcomputed tomography (µCT) analysis of the femurs of 12-week-old female and male mice revealed a significant decrease in trabecular bone mass of *SREBP2*^*ΔM*^ mice relative to control mice (Fig. 1a, b, Fig. S1c, d). In contrast, cortical parameters, including cortical bone volume/tissue volume and porosity, were comparable between the two groups (Fig. 1c, d, Fig. S1e, f). Bone histomorphometric analysis of the femur indicated that the trabecular osteoclast numbers (N. Oc/B.Pm), surface area (Oc. S/BS), and osteoclastic bone resorption activity (eroded surface, ES/BS) were significantly higher in *SREBP2*^*ΔM*^ mice than in control mice (Fig. 1e, f, Fig. S1g, h).

There was no significant difference in the mineral apposition rate (MAR) and bone formation rate (BFR/BS) between *SREBP2*^*ΔM*^ mice and *SREBP2*^*WT*^ mice (Fig. 1g, h). However, MAR shows a trend towards a decrease in *SREBP2*^*ΔM*^ mice compared to control mice (Fig. 1h). Consistently, C-terminal telopeptide of type I collagen (CTX), a bone resorption marker, was significantly increased in *SREBP2*^*ΔM*^ mice compared to control mice while procollagen type I N-terminal propeptide (P1NP), a bone formation marker, showed no significant difference between *SREBP2*^*ΔM*^ mice and *SREBP2*^*WT*^ mice (Fig. 1i), suggesting that SREBP2 deficiency in myeloid cells may have a limited effect on bone formation. Thus, our results suggest that myeloid cell-specific deletion of SREBP2 decreases bone mass through enhanced osteoclastogenesis in vivo.

### SREBP2 inhibits RANKL-induced osteoclastogenesis

**Fig. 2:**
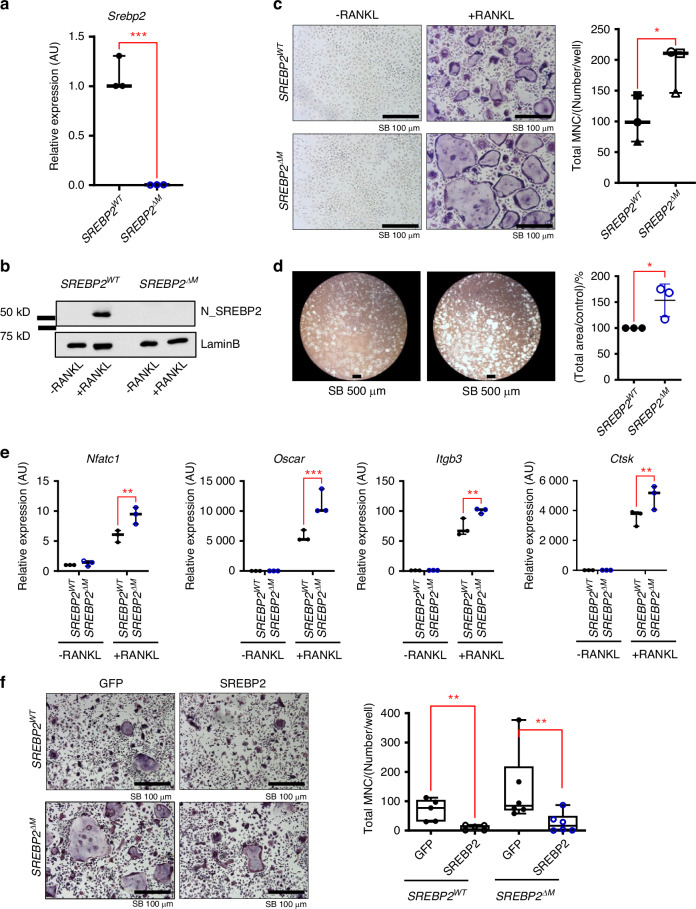
SREBP2 is a negative regulator of osteoclastogenesis. **a** *Srebp2* mRNA levels were assessed by qPCR from *SREBP2*^*WT*^ and *SREBP2*^*∆M*^ BMMs. **b–e** *SREBP2*^*WT*^ and *SREBP2*^*∆M*^ BMMs cultured with RANKL (50 ng/mL) for three days. **b** Immunoblots of nuclear lysates using SREBP2 and Lamin B antibodies. **c** Cells were TRAP-stained (left) and counted for 3 or more nuclei (right). Scale bars: 100 µm. **d** Bone resorption activity wase assessed by culturing *SREBP2*^*WT*^ and *SREBP2*^*∆M*^ BMMs with RANKL (50 ng/mL). Scale bars: 500 µm. **e** Osteoclast marker genes were assessed after RANKL (50 ng/mL) stimulation for 3 days. **f** *SREBP2*^*WT*^ and *SREBP2*^*∆M*^ BMMs transduced with adenoviruses harboring GFP control or FLAG_N_SREBP2 (SREBP2) were cultured with RANKL (50 ng/mL) (left). Scale bars: 100 µm. TRAP-positive cells with 3 or more nuclei were counted (right). All data are shown as median and interquartile range. n.s., not significant; *P < 0.05; **P < 0.01; ***P < 0.001 by paired *t* test (**a**, **c**, **d**) or two-way ANOVA with multiple comparisons (**e**, **f**). All data are from at least 3 independent experiments

**Fig. 3:**
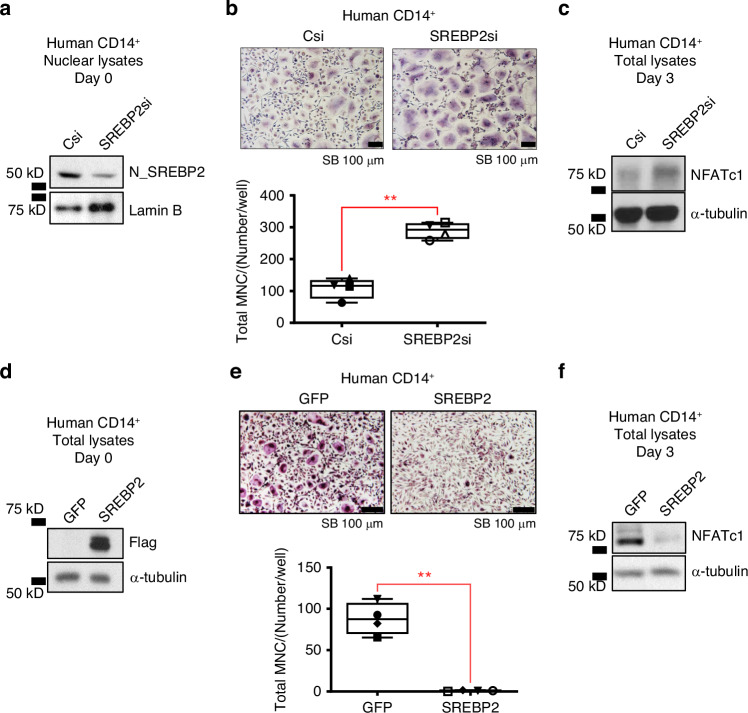
SREBP2 negatively regulates osteoclastogenesis in human CD14^+^ cells. **a**–**c** Human CD14^+^ monocytes were nucleofected with control siRNA (Csi) or SREBP2-targeted siRNA (SREBP2si). **a** Immunoblots of nuclear lysates using SREBP2 and Lamin B antibodies before RANKL stimulation. **b** Nucleofected cells cultured with RANKL (40 ng/mL) were TRAP-stained (upper) and counted for 3 or more nuclei (lower). Scale bars: 100 µm. **c** Immunoblots of total lysates at day 3 of RANKL (40 ng/mL) stimulation using NFATc1 and α-tubulin antibodies. **d**–**f** Human CD14^+^ monocytes were transduced with adenoviral particles encoding GFP control (GFP) or FLAG-N-SREBP2 (N-SREBP2). **d** Immunoblots of total lysates using Flag and α-tubulin antibodies before RANKL stimulation. **e** Transduced cells cultured with RANKL (40 ng/mL) were TRAP-stained and counted for 3 or more nuclei. Scale bars: 100 µm. **f** Immunoblots of total lysates at day 3 of RANKL (40 ng/mL) stimulation using NFATc1 and α-tubulin antibodies. All data are shown as median and interquartile range. **P < 0.01 by paired *t* test (**b**, **e**). All data are from at least 3 independent experiments

We next tested whether SREBP2 is involved in the regulation of RANKL-induced osteoclast differentiation by evaluating the effect of SREBP2 deletion in myeloid lineage cells on osteoclastogenesis. Bone marrow-derived macrophages (BMMs) were prepared from 12-week-old female *SREBP2*^*ΔM*^ mice and littermate control mice and cultured with M-CSF and RANKL for three days to differentiate into multinucleated osteoclasts. As expected, *Srebp2* expression was significantly reduced in SREBP2-deficient cells compared to control cells (Fig. 2a). This effect on SREBP2 deficiency was correlated with a significant reduction of nuclear (N)- SREBP2 in SREBP2-deficient cells relative to control cells (Fig. 2b). Consistent with our in vivo findings, RANKL-induced osteoclast differentiation was accelerated in SREBP2-deficient cells compared to control cells, as measured by the formation of TRAP^+^ multinucleated cells (Fig. 2c). The number of large osteoclasts (more than 20 nuclei) was significantly higher in SREBP2-deficientt cells compared to control cells (Fig. S2). When bone resorption by osteoclasts was assessed with calcium phosphate-coated plates, SREBP2 deficiency increased the area of resorption pits (Fig. 2d). Consistently, the expression of osteoclast-specific genes, including integrin beta 3 (*Itgb3*), and osteoclast-associated receptor (*Oscar*), was also elevated by SREBP2 deficiency (Fig. 2e). To corroborate our findings, we transduced control and SREBP2-deficient cells with adenoviral particles encoding N-SREBP2 or GFP. Overexpression of N-SREBP2 in control cells significantly suppressed RANKL-induced osteoclastogenesis. Moreover, reconstitution of SREBP2 in SREBP2-deficient cells suppressed enhanced osteoclastogenesis, indicating that SREBP2 deficiency is responsible for accelerated osteoclastogenesis in *SREBP2*^*ΔM*^ cells (Fig. 2f). To test whether human SREBP2 plays a similar function in RANKL-induced osteoclastogenesis in human cells, SREBP2 was knocked down in human CD14^+^ monocytes by small interfering RNAs (siRNAs), which enhanced RANKL-induced osteoclast differentiation and NFATc1 expression relative to control cells (Fig. 3a–c). Human CD14^+^ cells were transduced with adenoviral particles encoding N-SREBP2 or GFP. Decreased RANKL-induced osteoclastogenesis and NFATc1 were observed in N-SREBP2 overexpressed cells compared to control cells (Fig. 3d–f). Taken together, our results support that SREBP2 is a negative regulator of osteoclastogenesis in both mouse and human cells.

### SREBP2 regulates inflammatory bone destruction

**Fig. 4:**
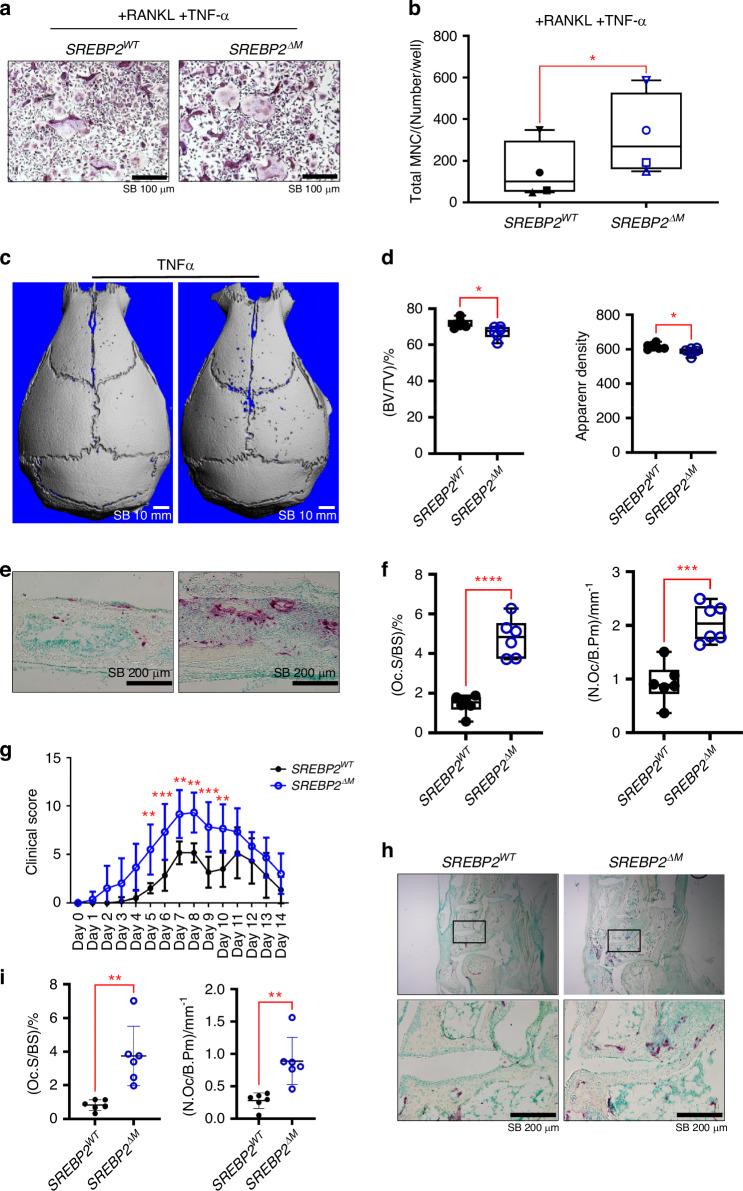
SREBP2 protects mice from inflammatory bone destruction. **a**, **b** Osteoclastogenesis assay. *SREBP2*^*WT*^ and *SREBP2*^*∆M*^ BMMs were cultured with RANKL (20 ng/mL) plus TNF-α (20 ng/mL). **a** Representative images of TRAP staining. Scale bars: 100 µm. **b** Quantification of TRAP-positive cells with 3 or more nuclei. **c**–**f** TNF-α-induced supracalvarial bone loss model (*n* = 6 per group). **c** Representative 3D images. **d** μCT analyses for bone volume/tissue volume (BV/TV) and apparent density. **e** Representative images for TRAP staining of histological sections of calvaria. Scale bars: 200 µm. **f** The osteoclast surface per bone surface (Oc. S/BS) and the number of osteoclasts per bone perimeter (N. Oc/B.Pm) were quantified. **g**–**i** K/BxN serum-induced arthritis model (*n* = 6 per group). **g** Time course of clinical score. **h** Representative TRAP staining of histological sections of tarsal bones. Scale bars: 200 µm. **i** The osteoclast surface per bone surface (OcC. S/BS) and the number of osteoclasts per bone perimeter (N. Oc/B.Pm) were quantified. All data are shown as median and interquartile range. n.s., not significant; *P < 0.05; **P < 0.01; ***P < 0.005; ****P < 0.001 by two-tailed unpaired *t* test (**b**, **d**, **f**, **i**), or 2-way repeated measures ANOVA with multiple comparisons (**g**)

Given that osteoclast-mediated bone erosion is a key clinical feature of RA,^18^ those findings led us to investigate the effect of SREBP2 in inflammatory bone destruction. Inflammatory cytokines, such as TNFα, promote osteoclastogenesis and play an important role in the pathogenesis of inflammatory osteolysis.^19^ mRNA and protein expression of SREBP2 increased in a time-dependent manner when cells were treated with TNF-α in combination with RANKL (Figs. S3a, b). To test the role of SREBP2 in inflammatory bone destruction, we first investigated whether SREBP2 is involved in the TNFα-mediated enhancement of osteoclastogenesis. BMMs from control and *SREBP2*^*ΔM*^ mice were cultured with an additional supplementation of TNFα. Intriguingly, SREBP2 deficiency significantly enhanced osteoclastogenesis by TNFα and RANKL co-treatment compared with control cells (Fig. 4a, b), suggesting that SREBP2 deficiency enhances osteoclast activity under inflammatory conditions. To further investigate the pathological importance of SREBP2 in regulating bone resorption in vivo, we investigated the role of SREBP2 in the TNFα-induced supracalvarial osteolysis model. TNFα administration over calvaria increased bone resorption pit area in *SREBP2*^*ΔM*^ mice compared to littermate control mice (Fig. 4c). µCT analysis revealed a significant decrease in calvaria bone volume/tissue volume and apparent density of *SREBP2*^*ΔM*^ mice compared to control mice (Fig. 4d). Moreover, the histomorphometric analysis demonstrated significant increases in Oc. S/BS and N. Oc/B.Pm in *SREBP2*^*ΔM*^ mice relative to littermate control mice (Fig. 4e, f), suggesting that inflammatory osteolysis is enhanced under SREBP2 deficiency.

To gain insight into the underlying mechanisms of pathological bone loss in RA, we tested the effects of enhanced osteoclastogenesis by SREBP2 deficiency in a K/BxN serum-induced arthritis model.^20^ *SREBP2*^*ΔM*^ mice showed a significant increase in inflammatory clinical score (Fig. 4g). However, histological assessment of the ankle joints on day 14 indicated that the resolution of inflammation was comparable between control and *SREBP2*^*ΔM*^ mice (Fig. S4a, b). Intriguingly, significant increases in Oc. S/BS and N. Oc/B.Pm were observed in the tarsal bone of *SREBP2*^*ΔM*^ mice (Fig. 4h, i). To further test the role of SREBP2 in inflammatory responses, BMMs from *SREBP2*^*ΔM*^ and control mice were stimulated with lipopolysaccharide. There were no statistically significant changes in the induction of inflammatory cytokines between *SREBP2*^*ΔM*^ cells and control cells (Fig. S5). Taken together, our findings indicate a protective role of SREBP2 in inflammation-associated pathological bone loss.

### SREBP2 increases at the late stage of osteoclast differentiation

**Fig. 5:**
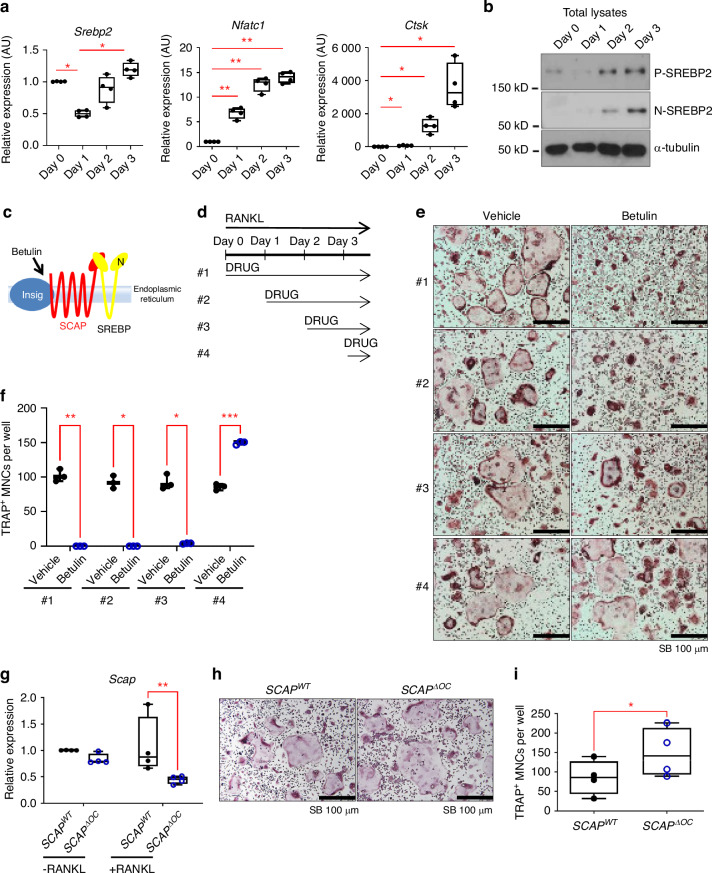
SCAP/SREBPs inactivation in late stage accelerates osteoclast differentiation. **a** *Srebp2, Nfatc1 and Cathepsin K* mRNA levels were assessed from 8-week-old female C57/BL6 mouse BMMs cultured with RANKL (50 ng/mL) for indicated days. **b** Immunoblots of total lysates from indicated days of culture after RANKL (50 ng/mL) stimulation using SREBP2 and α-tubulin antibodies. **c** Schematic showing the action of Betulin. **d** Schematic of drug treatment for experiments, #1 indicating drug treated since Day 0 of the culture (concurrent with RANKL), #2 from Day 1 of the culture, #3 from Day 2 of the culture, #4 from Day 3 of the culture. In case of #4, the drugs were incubated for 6 h. **e**, **f** 0 and 1 µg/mL of betulin were tested for four treatment schedules. **e** Representative images of TRAP-stained cells. **f** Numbers of TRAP-positive osteoclasts per well. **g** *Scap* mRNA levels were assessed by qPCR from *SCAP*^*WT*^ and *SCAP*^*∆OC*^ osteoclasts at day 3 of RANKL treatment. **h** Representative images of TRAP-stained cells. **i** Numbers of TRAP-positive osteoclasts from *SCAP*^*WT*^ and *SCAP*^*∆OC*^ are shown. All data are shown as median and interquartile range. n.s., not significant; *P < 0.05; **P < 0.01; ***P < 0.005; ****P < 0.001 by two-tailed unpaired *t* test (**i**), one-way (**a**) or two-way ANOVA with multiple comparisons (**f**, **g**). All data are from at least 3 independent experiments

To understand the role of SREBP2 during osteoclastogenesis, we examined the expression of SREBP2 mRNA and protein during osteoclastogenesis. *Srebp2* mRNA was diminished at day one after RANKL stimulation and then increased in a time-dependent manner in control BMMs, compared to the linear increase of *Nfatc1* and *Cathepsin K* (Fig. 5a). Consistently, the induction of total (P) and nuclear (N) SREBP2 proteins was observed two days after RANKL stimulation (Fig. 5b). Our results demonstrated that RANKL activated SREBP2 at the late stage of osteoclastogenesis.

Previous reports have established that the inhibition of SREBPs using pharmacological agents in the early stage of osteoclastogenesis inhibited osteoclast differentiation.^15–17^ To further examine the discrepancy, we tested the effects of the blockade of the SCAP/SREBPs axis on different stages of osteoclast differentiation. Betulin blocked the cleavage of SREBPs by promoting the interaction between Insig-1, a negative regulator, and SCAP (Fig. 5c).^21^ BMMs were treated with betulin in the different stages of osteoclastogenesis (treatment schedule #1-4) as shown in Fig. 5d. Consistent with previous observations showing a positive role of SCAP/SREBPs in osteoclastogenesis, the treatment of betulin at the same time of RANKL stimulation inhibited osteoclastogenesis in (Fig. 5e, f). The suppressive effect of betulin on osteoclastogenesis was attenuated when cells were treated at one day (#2) or two days (#3) after RANKL stimulation. Strikingly, the addition of betulin for 6 h at the late stage of osteoclastogenesis significantly enhanced osteoclastogenesis (#4). In addition, BMMs were also treated with PF429242, which inhibits S1P, a protease for activating SREBPs (Fig. S6a) in the different stages of osteoclastogenesis. Consistent with the observation made with betulin, late-stage treatment PF429242 enhanced osteoclastogenesis, with reduced nuclear translocation of N-SREBP2 and increased protein levels of integrin beta 3 (Fig. S6b–d). Consistently, osteoclast differentiation from human CD14^+^ monocytes was accelerated when PF429242 was treated at the late stage of osteoclastogenesis (Fig. S6e) with reduced translocation of N-SREBP2 into the nucleus (Fig. S6f).

To further examine the effect of SCAP/SREBPs complex inhibition at the late stage of osteoclastogenesis in vivo, we generated Cathepsin K expressing cell-specific SCAP-deficient mice by crossing Cathepsin K-Cre mice with SCAP^fl/fl^ mice (SCAP^fl/fl^ CtsK-Cre, referred to as SCAP^ΔOC^ mice). BMMs were prepared from 10-week-old male SCAP^ΔOC^ mice and control littermates and cultured with M-CSF and RANKL for three days to differentiate into multinucleated osteoclasts. As expected, *Scap* expression was reduced in Cathepsin K expressing cell-specific SCAP-deficient cells compared to control cells when they were treated with RANKL for three days (Fig. 5g). Consistent with the observation made with betulin and PF429242 at the late stage of osteoclastogenesis, RANKL-induced osteoclast differentiation was accelerated in Cathepsin K expressing cell-specific SCAP-deficient cells compared to control cells, as measured by the formation of TRAP^+^ multinucleated cells (Fig. 5h, i). Therefore, our findings suggest that inhibiting SREBPs at different stages may result in a differential effect on osteoclastogenesis, and SREBP2 inhibits osteoclastogenesis in the late stage of osteoclastogenesis.

### SREBP2-regulated osteoclastogenesis is independent of cholesterols

**Fig. 6:**
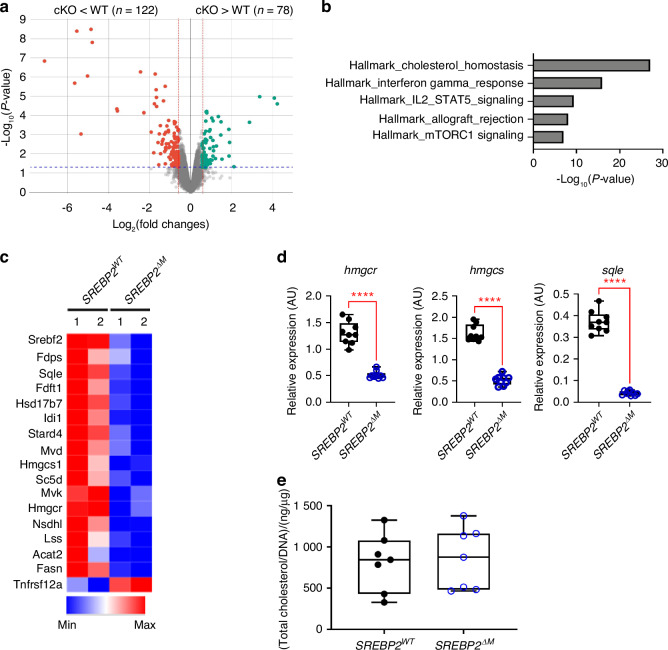
Total mRNA sequencing reveals differentially regulated genes in SREBP2 deficient osteoclasts. **a** Volcano plot of RNA-sequencing analysis of differentially expressed genes. Red dots represent significantly down-regulated and green dots represent up-regulated genes (*P* < 0.05, fold changes >1.5). **b** Gene Set Enrichment Analysis (GSEA) for pathways are shown. **c** Heatmap of representative cholesterol homeostasis genes from *SREBP2*^*WT*^ and *SREBP2*^*∆M*^ BMMs stimulated with RANKL (50 ng/mL) for 3 days. **d** *Hmgcr*, *Hmgcs*, and *Sqle* mRNA levels were assessed from *SREBP2*^*WT*^ and *SREBP2*^*∆M*^ BMMs stimulated with RANKL (50 ng/mL) for 3 days. **e** *SREBP2*^*WT*^ and *SREBP2*^*∆M*^ BMMs stimulated with RANKL (50 ng/mL) for 3 days were subjected to intracellular cholesterol measurement. Relative intracellular cholesterols in *SREBP2*^*WT*^ and *SREBP2*^*∆M*^ osteoclasts measured by Amplex Red cholesterol assay kit (*n* = 7). All data are shown as median and interquartile range. n.s., not significant; ****P < 0.000 1 by two-tailed unpaired *t* test (**d**, **e**). All data are from two biological replicates (**a**–**c**) and at least 3 independent experiments (**d**, **e**)

To gain an insight into the mechanisms by which SREBP2 inhibits osteoclastogenesis, we performed RNA sequencing (RNA-seq) analyses of osteoclasts from *SREBP2*^*ΔM*^ mice and littermate control mice. Two hundred genes were differentially regulated (*P* < 0.05, fold changes >1.5) between SREBP2-deficient osteoclasts and control osteoclasts: 78 genes were upregulated, and 122 genes were downregulated in SREBP2-deficient osteoclasts relative to control osteoclasts (Fig. 6a). Gene set enrichment analysis (GSEA) revealed that the pathways enriched in DEGs include cholesterol homeostasis, IFN-gamma response, IL2_STAT5 signaling, allograft rejection, and mTORC1 signaling (Fig. 6b). SREBP2 is a key transcription factor for cholesterol biosynthesis pathway.^22^ mRNA levels of *Hmgcr*, *Hmgcs*, and *Sqle* were reduced in SREBP2 deficient osteoclasts compared to control osteoclasts (Fig. 6c) and were confirmed by qPCR (Fig. 6d). While intracellular cholesterol levels were increased during RANKL-induced osteoclastogenesis in both mouse and human cells (Fig. S7a, b), intracellular cholesterol levels were comparable between SREBP2-deficient and control osteoclasts (Fig. 6e). Consistently, the labeling of intracellular free cholesterols with filipin, a fluorescent polyene antibiotic that binds to cholesterols, was also minimally affected by SREBP2 deficiency (Fig. S7c). Taken together, our results suggest that SREBP2 deficiency does not deplete cholesterols in osteoclasts and regulates osteoclasts independently of its conventional function as a regulator of cellular cholesterol synthesis.

### SREBP2 down-regulates RANKL-induced osteoclastogenesis via IRF7

**Fig. 7:**
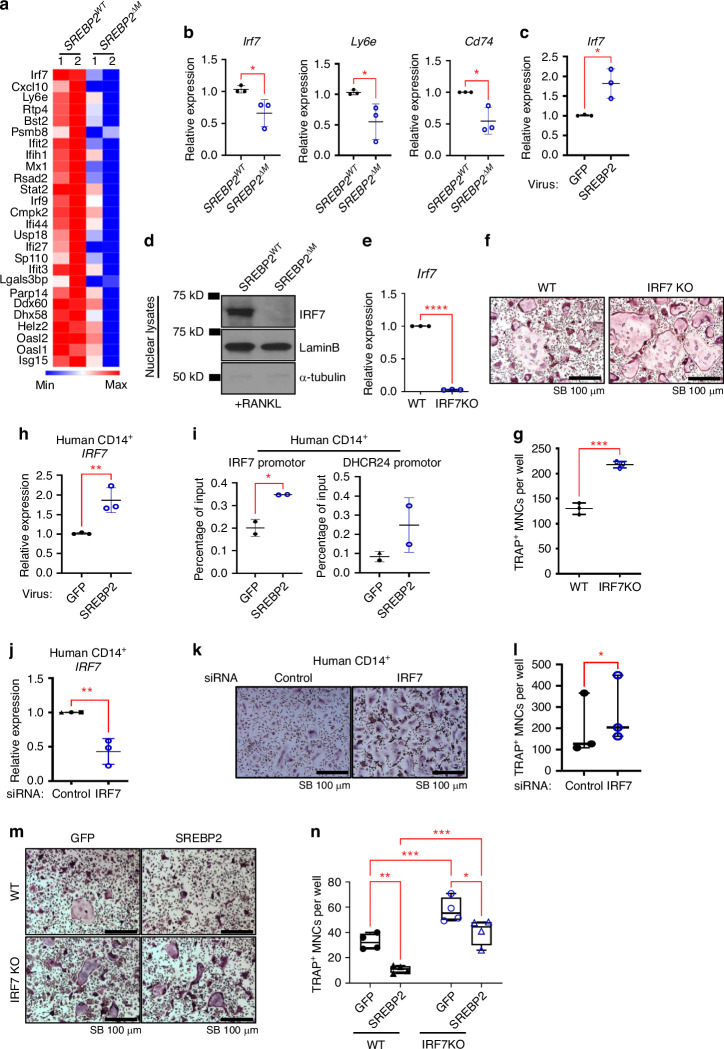
SREBP2 regulates IRF7. **a** Heatmap of representative interferon gamma response genes from *SREBP2*^*WT*^ and *SREBP2*^*∆M*^ BMMs stimulated with RANKL (50 ng/mL) for 3 days. **b** *Irf7*, *Ly6e*, and *Cd74* mRNA levels were assessed from *SREBP2*^*WT*^ and *SREBP2*^*∆M*^ BMMs stimulated with RANKL (50 ng/mL) for 3 days. **c** *Irf7* mRNA levels were assessed after adenoviral overexpression of GFP control or FLAG-N-SREBP2 (SREBP2) in mouse BMMs stimulated with RANKL (50 ng/mL). **d** Immunoblots of nuclear lysates from day 3 of RANKL (50 ng/mL) stimulation using anti-IRF7, SREBP2, Lamin B, and α-tubulin antibodies. **e** *Irf7* mRNA levels were assessed from 8-week-old female wildtype and IRF7 knockout BMMs. **f** Representative images of TRAP staining of wildtype and IRF7 knockout BMMs cultured with RANKL (50 ng/mL). Scale bars: 100 µm. **g** Quantification of TRAP-positive cells with 3 or more nuclei. RAP-stained Wildtype and IRF7 knockout BMMs cultured with RANKL (50 ng/mL) were TRAP-stained (upper) and counted for 3 or more nuclei (lower). Scale bars: 100 µm. **h** *IRF7* mRNA levels were assessed after adenoviral overexpression of GFP control or FLAG-N-SREBP2 (SREBP2) in human CD14^+^ monocytes stimulated with RANKL (40 ng/mL). **i** IRF7 and DHCR24 promoters were assessed by ChIP-qPCR analyses from human CD14^+^ monocytes transduced with adenoviruses harboring GFP control (GFP) or FLAG-N-SREBP2 (SREBP2). 2 independent experiments were performed from 4 different human donors. **J–l** *IRF7* mRNA levels were assessed from human CD14^+^ monocytes nucleofected with control or IRF7 siRNAs (**j**). Human CD14^+^ monocytes nucleofected with control o*r IRF*7 siRNAs cells were TRAP-stained after RANKL (40 ng/mL) stimulation (**k**) and counted for 3 or more nuclei (**l**). Scale bars: 100 µm. **m–n** Osteoclastogenesis assay. Wildtype and IRF7 knockout BMMs transduced with adenoviruses harboring GFP control or FLAG_N_SREBP2 (SREBP2) were cultured with RANKL (50 ng/mL). **m** Representative images were shown. Scale bars: 100 µm. **n** TRAP-positive cells with 3 or more nuclei were counted. All data are shown as median and interquartile range. n.s., not significant; *P < 0.05; **P < 0.01; ***P < 0.001; ****P < 0.000 1 by paired *t* test (**b**, **c**, **e**, **g**, **h**, **i**) or two-way ANOVA with multiple comparisons (**n**). All data are from two (a, i) and at least 3 independent experiments (**b**–**h**)

Genes in IFN-gamma response were enriched in SREBP2-deficient cells (Fig. 7a), and *Irf7, Ly6e, and Cd74* mRNA expression were lesser in SREBP2-deficient cells compared to control cells (Fig. 7b). Given the well-established negative feedback actions of IFNs on RANKL-induced osteoclastogenesis,^6,23–25^ we wished to test the role of SREBP2 on IFN responses. IRF7 is a critical factor for producing type I IFNs.^26^ but the role of IRF7 in osteoclastogenesis is largely unknown. We analyzed its mRNA and protein kinetics during osteoclastogenesis. *Irf7* mRNA was induced on day one after RANKL stimulation and maintained its expression throughout osteoclast differentiation in control BMMs (Fig. S8a), and the nuclear localization of IRF7 proteins was observed at the later phase of osteoclastogenesis (Fig. S8b). To corroborate the regulation of IRF7 by SREBP2, BMMs or human CD14^+^ cells were transduced with adenoviral particles encoding SREBP2 or GFP. Overexpression of SREBP2 enhanced IRF7 expression in BMMs (Fig. 7c) and human CD14^+^ cells (Fig. 7h). Consistent with our transcriptomic analysis, IRF7 protein was diminished in SREBP2-deficient cells compared with control cells (Fig. 7d). To test the role of IRF7 in osteoclastogenesis, BMMs from IRF7 global knockout mice and the age-and sex-matched wild type (WT) control mice were cultured with M-CSF and RANKL (Fig. 7e). Strikingly, IRF7 deficiency promoted in vitro osteoclastogenesis (Fig. 7f, g). The histomorphometric analysis also showed that the osteoclast number of femurs was significantly higher in IRF7 global knockout mice compared to that of control mice (Fig. S9a, b), suggesting that IRF7 deficiency increased osteoclastogenesis. However, bone mass in 9-week-old female IRF7 global mice was comparable to that of control mice (Fig. S9c, d). To identify the mechanisms by which SREBP2 regulates the expression of IRF7, we performed chromatin immunoprecipitation (ChIP) of SREBP2 in SREBP2 overexpressed cells. We observed direct binding of SREBP2 to the promoters of IRF7 and DHCR24, an SREBP2 target gene (Fig. 7i).

To further test the role of IRF7 in osteoclasts, IRF7 was knocked down in human CD14^+^ cells using siRNAs against IRF7 and controls (Fig. 7j). IRF7 knock-down (KD) also increased human osteoclastogenesis (Fig. 7k, l). We next tested whether SREBP2-mediated regulation of IRF7 contributes to enhanced osteoclast differentiation in SREBP2-deficient cells. BMMs from control and IRF7-deficient mice were transduced with adenoviral particles encoding GFP or SREBP2. Forced expression of SREBP2 by adenoviral transduction significantly suppressed osteoclastogenesis in control cells and IRF7 deficient cells. However, osteoclastogenesis in control cells with SREBP2 overexpression was significantly lower than that of IRF7-deficient cells with SREBP2 overexpression (Fig. 7m, n). Conversely, enhanced osteoclastogenesis in SREBP2 deficient cells was suppressed by overexpression of IRF7 (Fig. S10a, b). Collectively, our results suggest that SREBP2 is an upstream regulator and IRF7 is one of the major downstream effector molecules in suppressing osteoclastogenesis (Fig. S10c).

### The SREBP2-IRF7 axis regulates 26 overlapping genes

**Fig. 8:**
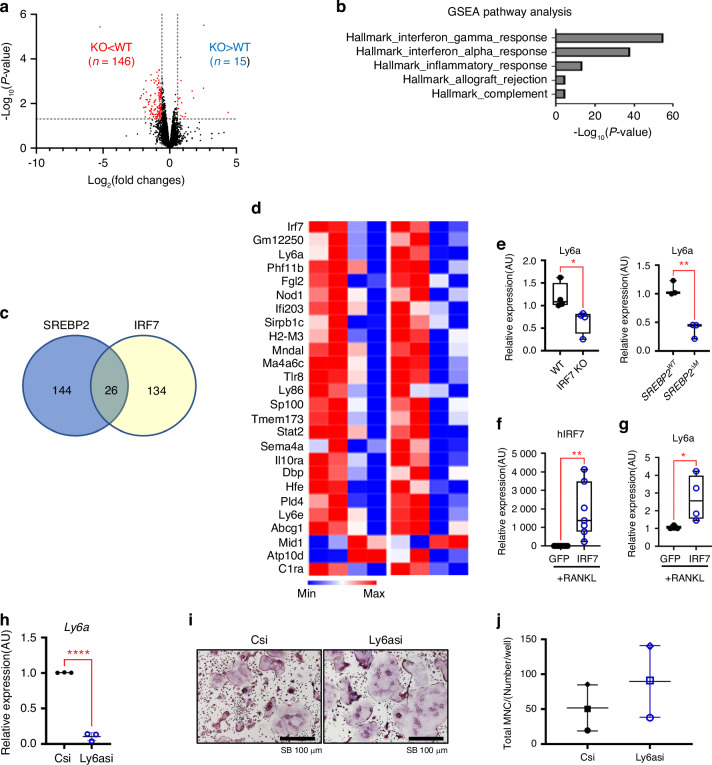
The SREBP2-IRF7 axis regulates 26 overlapping genes. **a** Volcano plot of RNA-seq analysis of differentially expressed genes (DEGs). Blue dots represent significantly differentially regulated genes (*P* < 0.05, fold changes >1.5). **b** Gene Set Enrichment Analysis (GSEA) of DEG in 7 a. **c** Venn diagram showing genes commonly regulated by SREPB2 and IRF7. **d** Heatmap of overlapped genes in DEGs shown in 5 a and 7 a. **e** *Ly6a* mRNA levels were assessed in wildtype, IRF7 knockout, *SREBP2*^*WT*^, and *SREBP2*^*∆M*^ BMMs after 3 days of RANKL (50 ng/mL) treatment. **f** *IRF7* mRNA levels were assessed after adenoviral overexpression of GFP control or 3xHA_GFP_IRF7 (IRF7) in mouse BMMs stimulated with RANKL (50 ng/mL). **g** *Ly6a* mRNA levels were assessed after adenoviral overexpression of GFP control or 3xHA_GFP_IRF7 (IRF7) in mouse BMMs stimulated with RANKL (50 ng/mL). **h** *Ly6a* mRNA levels were assessed from control siRNAs (Csi) or ly6a siRNAs (Ly6asi) transfected BMMs. **i** Osteoclastogenesis assay. Csi or Ly6asi transfected BMMs were cultured with RANKL (50 ng/mL). Representative images were shown. Scale bars: 100 µm. **j** TRAP-positive cells with 3 or more nuclei were counted. All data are shown as median and interquartile range. n.s., not significant; *P < 0.05; **P < 0.01 by paired *t*-test (e,f,g,h,j). All data are from two biological replicates (**a**–**d**) or at least 3 independent experiments (**e**–**h**, **j**)

To further elucidate the mechanisms by which the SREBP2-IRF7 axis regulates osteoclastogenesis, we analyzed the transcriptome of WT and IRF7-deficient osteoclasts by performing RNA-seq. IRF7-deficiency significantly upregulated the expression of 15 genes and downregulated the expression of 146 genes (*P* < 0.05, Fold changes> 1.5) (Fig. 8a). GSEA pathway analysis revealed that genes related to IFN gamma and IFN alpha responses were significantly enriched in differentially regulated genes between WT and IRF7-deficient osteoclasts (Fig. 8b). Although IRF7 is a critical transcription factor for type I IFNs, we were unable to detect *Ifnb* mRNA in our system (data not shown). However, we found that forced expression of IRF7 induced *Ifnb* mRNA expression, albeit with very low expression (Fig. S11a), suggesting that IRF7 may use an alternative pathway to suppress osteoclastogenesis in addition to the IFN pathway. To determine the potential target genes in the SREBP2-IRF7 axis, we first identified 26 overlapping genes commonly regulated by SREBP2 and IRF7 in osteoclasts (Fig. 8c and Table S1). Among them, stem cell antigen (Sca1, also known as Ly6a) was upregulated by RANKL treatment in control osteoclasts and commonly downregulated in both IRF7-deficient osteoclasts and SREBP2-deficient osteoclasts (Fig. 8d). *Ly6a* mRNA was downregulated in IRF7-deficient cells and SREBP2-deficient cells by qPCR analysis (Fig. 8e), suggesting that Ly6a is a common downstream target of the SREBP2-IRF7 axis. By contrast, overexpression of IRF7 induced Ly6a expression in osteoclasts (Fig. 8f, g). Although the reduced expression of Ly6a by siRNAs in mouse BMMs enhanced osteoclastogenesis compared to the control (Fig. 8h–j), the difference was not statistically significant due to the large variation in the data. Our data suggest that the SREBP2-IRF7 axis may regulate negative feedback mechanisms by regulating IFN-stimulating genes and Ly6a and provides a threshold for excessive osteoclast-mediated bone loss (Fig. S11b).

## Discussion

Chronic inflammation breaks the fine balance between bone resorption by osteoclasts and bone formation by osteoblasts, leading to pathological bone loss.^27^ The tight regulation of osteoclasts is important for averting excessive bone destruction in pathological conditions, including chronic inflammation. Although several negative feedback mechanisms in osteoclasts have been proposed, the current understanding of the negative feedback mechanisms in osteoclasts is limited and focuses on the early stage of osteoclastogenesis. In this study, we have identified the SREBP2-IRF7 axis as a novel negative feedback loop in the late phase of osteoclastogenesis. Our in vitro and in vivo evidence using osteoclast precursor cell-specific deletion of SREBP2 demonstrated that ablation of SREBP2 accelerated both the differentiation and activity of osteoclasts, resulting in reduced bone mass in *SREBP2*^*ΔM*^ mice. Moreover, we also showed that SREBP2 deficiency promoted in vivo osteoclastogenesis and associated inflammatory bone destruction in both a TNF-α-induced supracalvarial bone loss model and a K/BxN serum-induced arthritic model. Taken together, our findings reveal the role of SREBP2 in suppressing osteoclastogenesis and protecting against inflammatory bone destruction.

Our study identifies SREBP2 as a negative regulator at the late phase of osteoclastogenesis. Balancing positive and negative feedback mechanisms during osteoclastogenesis warrants controlled osteoclast formation and activity. Our in vivo model of myeloid cell-specific deletion of SREBP2 displayed a reduced BV/TV and diminished osteoclast number per bone surface (Fig. 1). There was a trend towards a decrease and significant decrease in Tb.Th (trabecular thickness) in the femur of female and male *SREBP2*^*ΔM*^ mice compared to control mice, respectively, suggesting that SREBP2 deficiency may contribute to changes in bone mass by enhancing osteoclast activity and affecting trabecular thickness.

The activity of SREBP2 is regulated by SCAP. However, SCAP influences the activity of all three isoforms of SREBPs. Accordingly, betulin, which is a SCAP inhibitor, also suppresses the activity of all three isoforms of SREBPs. Intriguingly, the treatment with betulin had a biphasic effect on osteoclast formation (Fig. 5e, f). When betulin was used in the early phase of osteoclast formation, it suppressed the process. However, when betulin was applied in the late phase, it enhanced osteoclast formation. Consistently, cells from SCAP-Cathepsin K cre mice (Fig. 5g–i), which may mimic the inhibition of SCAP at the later phase of osteoclastogenesis, showed enhanced osteoclastogenesis compared to control cells, supporting that targeting SCAP activity at the later osteoclast stages promotes osteoclast differentiation. We found that SREBP1 expression was initially high but decreased during osteoclast formation, while SREBP2 expression was induced in the later stage of the process (Fig. 5a). Our data suggest that SREBP2 might be the primary target of betulin in the later stages of osteoclast formation. Of note, to directly test the role of SREBP2 in the later stage of osteoclastogenesis, we attempted to generate SREBP2 flox/flox Cathepsin K cre mice. However, SREBP2-deficient cathepsin K cre homozygote mice were not born, suggesting the critical role of SREBP2 in cathepsin K-positive cells. As SREBP1 was dominant in the early stage of osteoclast formation, it is likely that betulin primarily affects SREBP1 in the early phase of osteoclast formation. Our findings indicate that targeting SREBP1 may suppress osteoclast formation; however, further study is needed to determine the effect of SREBP1 on osteoclastogenesis. Taken together, these results suggest that despite constant SCAP expression, the activity of SREBPs is fine-tuned, and their effect on osteoclastogenesis is also tightly regulated.

The importance of cholesterol metabolism in osteoclasts has been well appreciated and cholesterols positively regulate osteoclastogenesis.^14^ The SCAP-SREBP2 axis regulates the expression of genes related to cholesterol biosynthesis.^11^ Indeed, gene expressions in the cholesterol biosynthesis pathway were significantly decreased in SREBP2-deficient BMMs. However, we found that myeloid cell-specific SREBP2 deficiency has little effect on the intracellular cholesterol level when they differentiate into osteoclasts in vitro. Similar results were observed in a previous publication investigating the intracellular cholesterol pool of SCAP-deficient BMMs; the contribution of cholesterol synthesis to total cholesterol pools was less than 5% in WT BMMs, and there were no significant changes in total cholesterol pools in SCAP-deficient BMMs despite the reduced cholesterol synthesis in SCAP-deficient BMMs.^28^ These results suggest that the SCAP-SREBP2 axis-mediated cholesterol synthesis has a limited effect on total cholesterol pools in BMMs due to the high influx of cholesterols. Thus, despite the pivotal role of SREBP2 in cholesterol synthesis pathway gene expression, it is possible that the modulatory role of SREBP2 in osteoclasts is the activation of a novel negative feedback pathway and is independent of its role in cholesterol biosynthesis.

Our results show that IRF7 contributes to the negative feedback loops that modulate osteoclast differentiation. IRF7 has been reported as the master regulator of type I IFN pathways and its involvement in IFN-β production has been well established.^26,29^ Higher expression of IRF7 and/or IFN-β is associated with a lower risk of bone metastasis in breast and prostate cancer,^30,31^ but the role of IRF7 in osteoclastogenesis, to our knowledge, has not been shown yet. IFNs are a well-established negative regulator of osteoclastogenesis^6,24,32^ and an inducer of IRF7.^29^ RANKL stimulation induces IFNβ production through the transcription factor c-FOS, a key transcription factor of osteoclastogenesis,^6^ and, in turn, IFNβ suppressed c-FOS expression at the early stage of osteoclastogenesis, leading to inhibiting osteoclast differentiation.^6,33,34^ IFNγ is also known as a negative regulator of osteoclast differentiation by inducing TRAF6-degradation downstream of RANK.^24^ and/or diminishing RANK expression.^35^ Accordingly, mice deficient in essential components of the IFN pathways including IFNAR1, STAT, IRF1, or IRF9, exhibited reduced bone density and increased osteoclastogenesis.^6,32,36^ A recent report has also shown that IFN-stimulated genes (ISGs) including guanylate-binding proteins (GBPs)^32^ and ISG15^37^ negatively regulate inflammatory bone loss. Our study showed that IRF7 is a negative regulator of osteoclast differentiation, emphasizing the role of ISGs in osteoclastogenesis. While IRF7 was minimally present in the early phase of osteoclasts, its expression was evident in the late phase of osteoclastogenesis and was induced by SREBP2, suggesting the synergistic activation of IRF7 by SREBP2 and IFN responses during osteoclastogenesis. IRF7-deficiency suppressed a subset of RANKL-induced ISGs. However, the levels of IFNβ in osteoclasts were very low even when IRF7 was induced, and thus, we were not able to detect IFNB1 mRNA in our system. Our results raise the possibility that SREBP2 may augment the induction of IRF7, which can be mediated by low levels of IFNs during osteoclastogenesis. Since emerging evidence showed that ISGs can directly affect osteoclastogenesis,^32,37^ it is important to further characterize the underlying mechanisms of IFN-mediated negative feedback loops. In addition to ISGs, we also represented Ly6a as one of downstream targets of the SREBP2-IRF7 regulatory circuit of osteoclasts, along with 25 other overlapped genes, and the role of 26 overlapped genes in osteoclasts will be further investigated in a future study.

Recent studies have highlighted novel roles of SREBPs in innate and adaptive immune responses. Immune cells bring in SREBPs-regulated lipid metabolism to control its effector function in natural killer cells and to induce trained immunity in T cells.^38,39^ In addition, SCAP/SREBPs can regulate type I IFN signaling and inflammasome in macrophages.^28,40^ and SREBP2 promotes inflammatory macrophage polarization by inducing inflammatory and IFN response genes in primary human macrophages.^13^ However, the effect of SREBP2 on inflammatory response using the genetic model has not been investigated up to date. Although SREBP2 deficiency further enhanced osteoclastogenesis under inflammatory conditions, the expression of inflammatory cytokines was not significantly different between *SREBP2*^*ΔM*^ cells and control cells after LPS stimulation. However, we haven’t tested all other inflammatory responses, and our experimental sample size was limited, potentially resulting in underpowered variation. Therefore, further research is necessary to make conclusive statements about the role of SREBP2 in inflammatory responses.

In our study, we demonstrated for the first time that the SREBP2-IRF7 axis provides negative feedback toward the maturation of fully functional osteoclasts and plays an important role in pathological bone destruction. These results changed the way we think about the cellular role of SREBP2 and introduced the concept that a transcription factor typically dedicated to a metabolic pathway can be repurposed by RANKL stimulation to assume direct bone remodeling, especially in chronic inflammatory arthritis.

## Materials and methods

### Mice

Lysozyme M promoter-driven Cre transgene on the C57/BL6 background mice (known as LysMcre mice) and *SREBP2*^*flox/flox*^ mice were purchased from The Jackson Laboratory. LysMcre mice were crossed with *SREBP2*^*flox/flox*^ mice to generate *Srebp2*^*fl/fl*^ *LysM-Cre* (referred to as *SREBP2*^*ΔM*^) and *Srebp2*^*+/+*^ *LysM-Cre* (referred to as *SREBP2*^*WT*^) mice. Cathepsin K promoter-driven Cre transgene on the C57/BL6 background mice (known as CtsKcre mice) were obtained from Dr. Takashi Nakamura.^41^ *SCAP*^*flox/flox*^ mice were also purchased from The Jackson Laboratory. CtsKcre mice were crossed with *SCAP*^*flox/flox*^ mice to generate *SCAP*^*fl/fl*^ *CtsK-Cre* (referred to as *SCAP*^*ΔOC*^ mice). IRF7 deficient mice were obtained from Dr. Deng.^26^ Oligonucleotide sequences for genotype OCR are in Table S2. All animals were housed in a specific pathogen-free environment in the Weill Cornell Medicine vivarium and all the experiments conformed to the ethical principles and guidelines approved by the Institutional and Animal Care and Use Committee of Weill Cornell Medical College and Sloan Kettering Cancer Institute (IACUC #2015-0065).

### Inflammatory bone destruction models

TNF-induced supracalvarial osteolysis model was performed as previously described. Briefly, recombinant human TNF-α was administrated daily injected at the dose of 40 µg/kg to calvarial periosteum of 12-week-old female control and *SREBP2*^*ΔM*^ mice for five consecutive days. For K/BxN serum-induced arthritis model, K/BxN serum was collected as previously described.^42^ In brief, 80 µL of K/BxN sera were administered intraperitoneally to 14-week-old C57/BL6 female mice on days 0 and 2 and the mice were euthanized on day 14 after daily monitoring and measuring the thickness of wrist and ankle joints using calipers. The thickness of joints (total of 4 per mouse) was summed up and described in the graph.

### Micro-CT and histomorphometry analysis

To analyze bone volume and architecture, μ-CT analysis was performed using micro-CT-35 instrument (Scanco Medical, Bruttisellen, Switzerland) as described previously,^43,44^ and all samples were included in the analysis conducted in a blinded manner. For μCT analysis, prior to decalcification, femurs were scanned by μCT, with an isotropic voxel resolution of 6 µm (μCT35, Scanco, Bruttisellen, Switzerland; 55kVp, 145μA, 600 ms integration time) to evaluate morphological changes in bone. Bone morphology in the femur was examined in two regions: the diaphysis and the metaphysis. For cortical bone, the volume of interest (VOI) encompassed cortical bone within a 100-slice section in the diaphysis. For trabecular bone, the VOI encompassed a 200-slice section in the metaphysis, proximal to the growth plate. To ensure exclusion of primary spongiosa in the growth plate, VOIs began 50 slices proximal to the median of the growth plate. Outcome parameters for cortical bone included thickness and tissue mineral density (TMD). Trabecular bone parameters included bone volume fraction (BV/TV), trabecular thickness (Tb.Th), trabecular spacing (Tb.Sp), and trabecular number (Tb.N.). 3D reconstructions were generated by stacking thresholded 2D images from the contoured region. To measure a bone formation rate, the dynamic bone labeling analysis was performed as previously described. In brief, calcein (Sigma) was dissolved in 2% NaHCO_3_ and administered twice at the dose of 10 mg/kg with a 5-day interval. The right femurs were fixed in 4% PFA for 4 days and decalcified for 2 days, followed by 10% sucrose solution overnight incubation at 4 °C. After embedding in Tissue-Tek OCT compound (Sakura Finetek), frozen section at 9 µm thickness was performed. After mice were euthanized, femurs, calvarial bones, hind paws, and ankles were collected for histologic assessment. The bones were fixed in 4% paraformaldehyde (PFA) for 4 days followed by decalcification in 10% EDTA (Sigma Aldrich) solution for 4 weeks. Paraffin embedded samples were subjected for 8–9 µm sectioning followed by TRAP staining and methyl green counterstaining (Vector Laboratories) for clear visualization of osteoclast and bone. Trabecular analyses were confined to the secondary spongiosa of femurs (excluding cortical bone), covering approximately 1.5 mm^2^ tissue area. For the supracalvarial osteolysis model, the analyses were restricted to the right parietal (where TNF-α injected), excluding frontal, interparietal, and occipital regions. For the K/BxN model, left tarsal bones were subjected to analyses, including the surfaces of cuneiforms, navicular, and cuboid. Multinucleated TRAP^+^ cells that adhered to the bone surface were considered osteoclasts. All measurements were performed using Osteometric software (Osteomeasure) following the standard procedure.^45^

### In vitro osteoclastogenesis

Mouse bone marrow macrophages (BMMs) were prepared as previously described.^43^ BMMs were committed to the pre-osteoclast lineage by treating them with 5% L929 cell supernatant, which served as a source of M-CSF conditioned medium (CM).^46^ In brief, flushed bone marrow cells from the femurs and tibiae were incubated for 1 min in ACK lysing buffer (Gibco) to remove red blood cells and cultured with CM for 3 days. Cultured cells were scraped, counted, and seeded at a density of 10^5^/mL in an appropriate plate (triplicate wells on a 96-well plate for TRAP staining, single to double wells on a 6-well plate for RNA, single to double wells on a 24-well plate for protein). 5% CM and RANKL (50 ng/mL, Peprotech) were added in α-MEM (Thermo Fisher Scientific) supplemented with 10% FBS (R&D systems), 1% glutamine (200 mmol/L, Thermo Fisher Scientific), and 1% pen/strep antibiotics (Thermo Fisher Scientific) to induce osteoclasts with every 2 days replenishment.

For human osteoclast differentiation assay, the procedures were followed by previously described method.^47^ This protocol was approved by the Hospital for Special Surgery Institutional Review Board (IRB #2019-0681). Briefly, peripheral blood mononuclear cells (PBMCs) were collected from blood leukocyte preparations purchased from the New York Blood Center by density gradient centrifugation with Ficoll (Thermo Fisher Scientific). Anti-CD14 magnetic beads (0.6 µL/10^6^ cells, Miltenyi Biotec) were used to select CD14^+^ monocytes. Cells were plated at a density of 10^6^/mL in an appropriate plate (triplicate wells on a 96-well plate for TRAP staining, single to double wells on a 6-well plate for RNA, single to double wells on a 24-well plate for protein). M-CSF (20 ng/mL, Peprotech) and RANKL (40 ng/mL, Peprotech) were added in α-MEM (Thermo Fisher Scientific) supplemented with 10% FBS (Hyclone, GE Healthcare Life Sciences) and 1% glutamine (200 mmol/L, Thermo Fisher Scientific) to induce osteoclasts. Cells seeded on the 96 plates were fixed and stained for TRAP using the Acid Phosphatase Leukocyte Diagnositic Kit (Sigma Aldrich). Multinucleated TRAP^+^ cells with 3 or more nuclei were considered as osteoclasts. For bone resorption pit assay, mouse BMMs were seeded at a concentration of 2 ×10^5^/mL in 96-well Corning Osteo Assay Surface Plates (Sigma). 5% CM and RANKL (50 ng/mL, Peprotech) were added in α-MEM supplemented with 10% FBS, 1% glutamine, and 1% pen/strep antibiotics to induce osteoclasts with every 2 days replenishment. Total culture days were 6 to 8 days for optimal resorption in the entire well. Cells were removed by 10% bleach solution followed by 1% toluidine blue staining for clear visualization of resorbed pits.

### Quantitative real-time PCR

RNeasy Mini Kit (Qiagen) with DNase treatment was used to prepare DNA-free RNA. 100–800 ng total RNA was reverse transcribed using the First Strand cDNA synthesis kit (Thermo Fisher Scientific) following the manufacturer’s protocol. Real-time PCR was performed in triplicate with Fast SYBR Green Master Mix (Applied Bisosystems) and ran on 7500 Fast Real-time PCR system (Applied Bisosystems). Human hypoxanthine guanine phosphoribosyl transferase (*Hprt*) and mouse TATA box-binding protein (*Tbp*) were used for housekeeping internal controls for human and mouse cells respectively. Oligonucleotide sequences are in Table S3.

### RNA-sequencing

Total RNA was prepared by an RNeasy Mini Kit with DNase treatment. 200 ng RNAs were used to construct mRNA libraries using TruSeq Stranded mRNA kits (Illumina). Multiplexed barcode adapters were ligated to the libraries following the manufacturer’s protocols. Samples that were passed quality control analysis by a Bioanalyzer 2100 (Agilent) were submitted to the Weill Cornell Medical College Genomics Resources Core Facility. Paired-end reads (50 × 2 cycles, ~75 × 10^6^ reads per sample) were obtained on an Illumina NovaSeq 6000.

### Immunoblotting

Whole cell lysates were collected using 1x Laemmli Sample Buffer (Bio-Rad), and nuclear lysates were prepared by Buffer A solution (10 mmol/L Hepes, 10 mmol/L KCl, 0.1 mmol/L EDTA, 0.1 mmol/L EGTA, 1 mmol/L DTT, 1x proteinase inhibitor cocktail). Then, boiled lysates were loaded onto 7.5% or 10% SDS-PAGE, followed by standard Western blotting protocols. Alpha-tubulin for whole cell lysates and Lamin B for nuclear lysates were used as loading control. Anti-SREBP2 (PA1-338) antibody was purchased from Thermo Fisher Scientific (Waltham, USA), anti-SREBP2 (ab30682) from Abcam (Cambridge, UK), and Lamin B (12987–1-AP) from Proteintech (Rosemont, USA).

### Cholesterol quantitation

An Amplex Red Cholesterol Assay Kit (Invitrogen) was used to perform cholesterol quantitation. In brief, 5 × 10^5^ cells were extracted with 100 µL of chloroform: isopropanol: NP-40/IGEPAL-CA-630 (7:11:0.1) in a Bioruptor (Diagenode) with 30 s on 30 s off on a high-power output for 10 cycles for homogenization. Then, samples were centrifuged at 13 000 × *g* for 10 min to remove insoluble materials. Next, organic phase was transferred to a new tube and air dried completely at 50 °C to remove chloroform and organic residues. Then, 1X reaction buffer (included in the kit) was added and further processed according to the manufacturer’s instructions. Fluorescence intensity was measured with Varioskan Flash Spectral Scanning (Thermo Scientific) and 5 × 10^5^ cells were subjected for DNA quantitation using a DNeasy Blood & Tissue Kit (Qiagen) for normalization.

### Fluorescence microscopy

BMMs from control and *SREBP2*^*ΔM*^ mice were cultured with M-CSF and RANKL for three days, and then, cells were fixed with 4% formaldehyde for 10 min. Next, cells were stained with Phalloidin-iFluor 568 for 20 min at room temperature, followed by Filipin (Sigma F9765) staining for 2 h at room temperature. Stained cells were viewed using the red and blue filters equipped in the ZEISS LSM 880 with Airyscan or Nikon Ni-E Eclipse microscope. Randomly chosen 5 fields with regions of interest (mature osteoclasts) were subjected to analysis with the QuPath (version 0.2.3) program for blue color intensity in osteoclasts.

### Adenoviral transduction

Recombinant adenoviral particles encoding human nuclear domain of SREBP2 with FLAG protein (Ad-CMV-2xFLAG-SREBP2) was custom built from Vector Biolabs and was also used in previous report.^13^ Recombinant adenoviral particles encoding human IRF7 with HA protein (Ad-GFP-h-IRF7.3xHA) was custom ordered from Vector Biolabs. Ad-CMV-GFP from Vector Biolabs was used as control adenoviral particles. For transduction experiment, mouse BMMs were seeded at a density of 2 ×10^5^ cells/mL on a 6 well plate with daily addition of M-CSF (40 ng/mL) for 2 days. Then, the complete media was exchanged to low serum media (2% FBS with no added glutamine and antibiotics) containing M-CSF (20 ng/mL), followed by adenoviral particles (150 MOI) addition. Human monocytes were seeded at a density of 2 × 10^6^ cells/mL on a 6 well plate with daily addition of M-CSF (40 ng/mL) for 4 days. Then, the complete media was exchanged to low serum media (2% FBS with no added glutamine) containing M-CSF (20 ng/mL), followed by adenoviral particles (30 MOI) addition. The plates were centrifuged at 1 600 r/min for 30 min at room temperature. After 12 h of transduction, fresh complete media was replaced and incubated for >8 h before further process. The green fluorescent was monitored for efficiency of transduction.

### RNA interference

0.4 nmol of siRNA oligonucleotides targeting SREBP2 (Dharmacon Cat#L-009549-00-0005) or Ly6a (Dharmacon Cat#L-063198-00-0005) were nucelofected in human CD14^+^ monocytes using a Nucleofector kit (Lonza) as previously described.^48^ Human Monocyte Nucleofector buffer and the AMAXA Nucleofector System (Lonza) program Y001 were used following the manufacturer’s instructions.

### Chromatin immunoprecipitation

ChIP experiments were performed as previously described. After cells were cultured with M-CSF and RANKL, cells were fixed with 1% formaldehyde (Thermo Scientific) for 5 mins. 0.125 mol/L glycine was added for quenching the formaldehyde residues. Next, cells were lysed/washed and sonicated sequentially using LB1, LB2, and LB3 (LB1, 50 mmol/L HEPES-KOH, pH 7.5, 140 mmol/L NaCl, 1 mmol/L EDTA, 10% glycerol, 0.5% NP-40 and 0.25% Triton X-100; LB2, 10 mmol/L Tris-HCl, pH 8.0, 200 mmol/L NaCl, 1 mmol/L EDTA and 0.5 mmol/L EGTA; LB3, 10 mmol/L Tris-HCl, pH 8.0, 100 mmol/L NaCl, 1 mmol/L EDTA, 0.5 mmol/L EGTA, 0.1% sodium deoxycholate and 0.5% N-lauroylsarcosine). Nuclear lysates were sonicated with Bioruptor using a cycle with 30 s on 30 s off on a high-power output for 8 cycles. The sonicated samples were centrifuged at 20 000 × *g* for 15 min at 4 °C. Supernatants were collected and 5% of supernatants were saved as input and DNA analysis and the rest was incubated overnight with 5 µg of anti-FLAG M2 antibody at 4 °C (Sigma Aldrich) on a rotator. After overnight incubation, the magnetic beads were washed in ice-cold condition as follow (once in low salt buffer, 150 mmol/L NaCl, 0.5% Na deoxycholate, 0.1% SDS, 1% Nonidet P-40, 1 mmol/L EDTA pH 8.0, 50 mmol/L Tris HCl pH 8.0; once in high salt buffer, 500 mmol/L NaCl, 0.5% Na deoxycholate, 0.1% SDS, 1% Nonidet P-40, 1 mmol/L EDTA pH 8.0, 50 mmol/L Tris HCl pH 8.0; once in LiCl wash buffer, 100 mmol/L LiCl, 0.5% Na deoxycholate, 0.1% SDS, 1% Nonidet P-40, 1 mmol/L EDTA pH 8.0, 50 mmol/L Tris HCl pH 8.0; once in TE wash buffer, 10 mmol/L Tris-HCl pH 8.0, 1 mmol/L EDTA, 50 mmol/L NaCl). Then, DNA was eluted with elution buffer (50 mmol/L Tris-HCl pH 8.0, 10 mmol/L EDTA, 1% SDS) with added 0.3 mol/L NaCl and incubated for overnight at 65°C. Next day, the samples were incubated with Rnase for an hour at 37 °C followed by Proteinase K incubation for 2 h at 55 °C. Then, the DNA was purified with PCR purification kit (Qiagen) and subjected for quantitative Real-Time PCR assays.

### Pathway analysis using GSEA

Gene set enrichment analysis (GSEA) is used for analyzing the differentially expressed genes.^49^ Hallmark gene sets in MSigDB (molecular signature data base) were used for the analysis. Pathways were ranked based on *P* values.

### Statistics

Data are presented as the mean ± SD of biological replicates. Animals for each experiment were randomly selected for further treatments. An unpaired two-tailed Student’s *t* test was used to assess differences between two groups. One-way or two-way analysis of variance with post hoc Bonferroni or Tukey’s test was used for analyses of multiple groups. All statistical tests were performed using GraphPad Prism 8 (GraphPad Software). A *P* value of less than 0.05 was considered statistically significant.

### Study approval

Animal experiments were performed in compliance with ethical regulations and approved by the Hospital for Special Surgery and Weill Cornell Medical College Institutional Animal Care and Use Committee (IACUC #2015-0065). The protocol for human osteoclast differentiation assay was approved by the Hospital for Special Surgery Institutional Review Board (IRB #2019-0681).

### Supplementary information

Supplemental material

## Data Availability

The bulk RNA-seq datasets that were generated by the authors as part of this study have been deposited in the Gene Expression Omnibus database with the accession code GSE172007. The data can be accessed via https://www.ncbi.nlm.nih.gov/geo/query/acc.cgi?acc=GSE172007.
