# Supplementary material for: SREBP2 restricts osteoclast differentiation and activity by regulating IRF7 and limits inflammatory bone erosion
Source: Bone Res. 2024 Aug 27;12:48. doi: 10.1038/s41413-024-00354-4 (PMC11350122; doi:10.1038/s41413-024-00354-4)
Supplement: Supplementary file 1 — Supplemental material [file 41413_2024_354_MOESM1_ESM.pptx]

## Slide 1
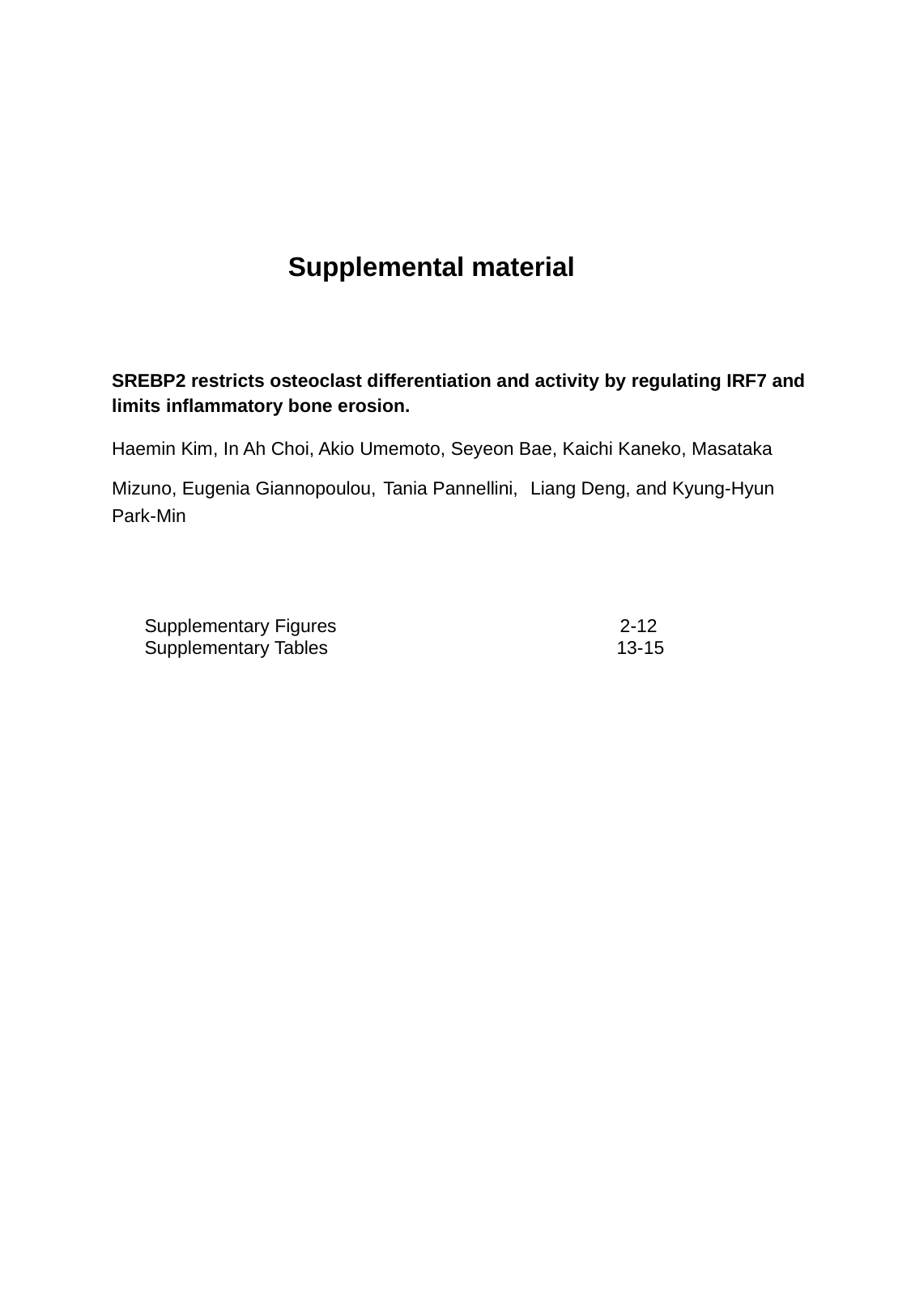

Supplemental material
SREBP2 restricts osteoclast differentiation and activity by regulating IRF7 and limits inflammatory bone erosion.
Haemin Kim, In Ah Choi, Akio Umemoto, Seyeon Bae, Kaichi Kaneko, Masataka Mizuno, Eugenia Giannopoulou, Tania Pannellini, Liang Deng, and Kyung-Hyun Park-Min
Supplementary Figures 2-12
Supplementary Tables 13-15

## Slide 2
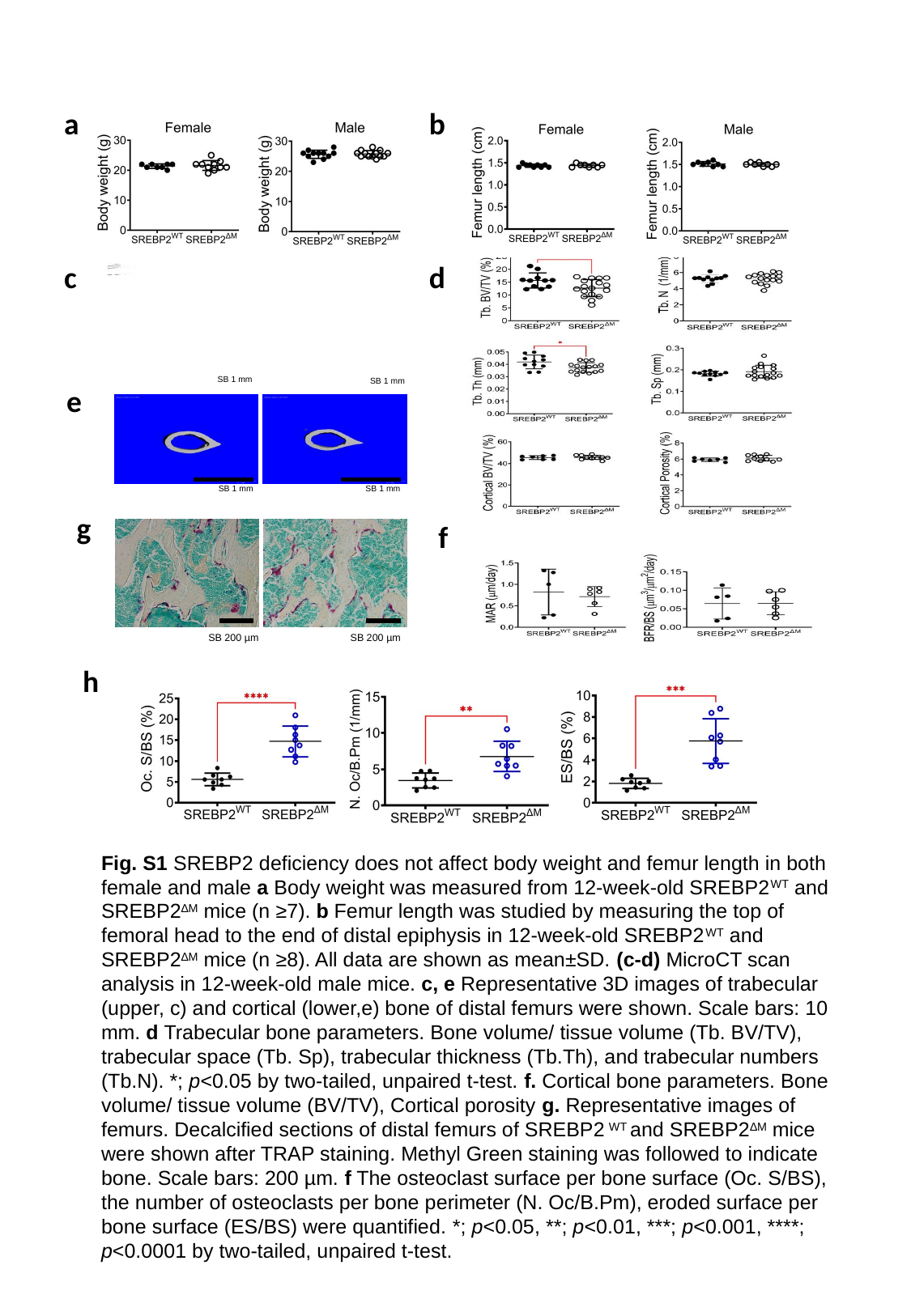

a
b
c
d
SB 1 mm
SB 1 mm
e
SB 1 mm
SB 1 mm
g
f
SB 200 µm
SB 200 µm
h
Fig. S1 SREBP2 deficiency does not affect body weight and femur length in both female and male a Body weight was measured from 12-week-old SREBP2WT and SREBP2∆M mice (n ≥7). b Femur length was studied by measuring the top of femoral head to the end of distal epiphysis in 12-week-old SREBP2WT and SREBP2∆M mice (n ≥8). All data are shown as mean±SD. (c-d) MicroCT scan analysis in 12-week-old male mice. c, e Representative 3D images of trabecular (upper, c) and cortical (lower,e) bone of distal femurs were shown. Scale bars: 10 mm. d Trabecular bone parameters. Bone volume/ tissue volume (Tb. BV/TV), trabecular space (Tb. Sp), trabecular thickness (Tb.Th), and trabecular numbers (Tb.N). *; p<0.05 by two-tailed, unpaired t-test. f. Cortical bone parameters. Bone volume/ tissue volume (BV/TV), Cortical porosity g. Representative images of femurs. Decalcified sections of distal femurs of SREBP2 WT and SREBP2∆M mice were shown after TRAP staining. Methyl Green staining was followed to indicate bone. Scale bars: 200 µm. f The osteoclast surface per bone surface (Oc. S/BS), the number of osteoclasts per bone perimeter (N. Oc/B.Pm), eroded surface per bone surface (ES/BS) were quantified. *; p<0.05, **; p<0.01, ***; p<0.001, ****; p<0.0001 by two-tailed, unpaired t-test.

## Slide 3
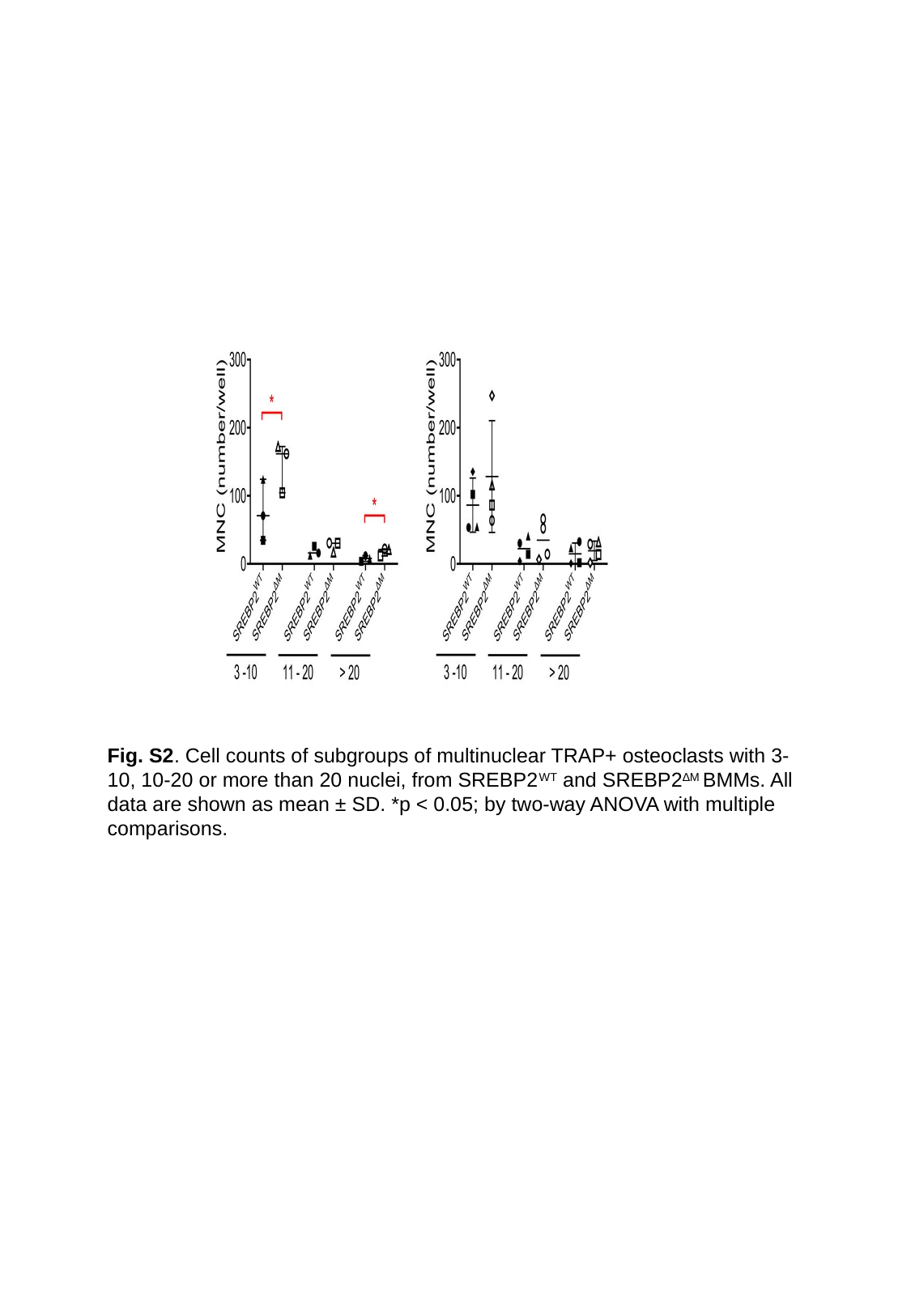

Fig. S2. Cell counts of subgroups of multinuclear TRAP+ osteoclasts with 3-10, 10-20 or more than 20 nuclei, from SREBP2WT and SREBP2∆M BMMs. All data are shown as mean ± SD. *p < 0.05; by two-way ANOVA with multiple comparisons.

## Slide 4
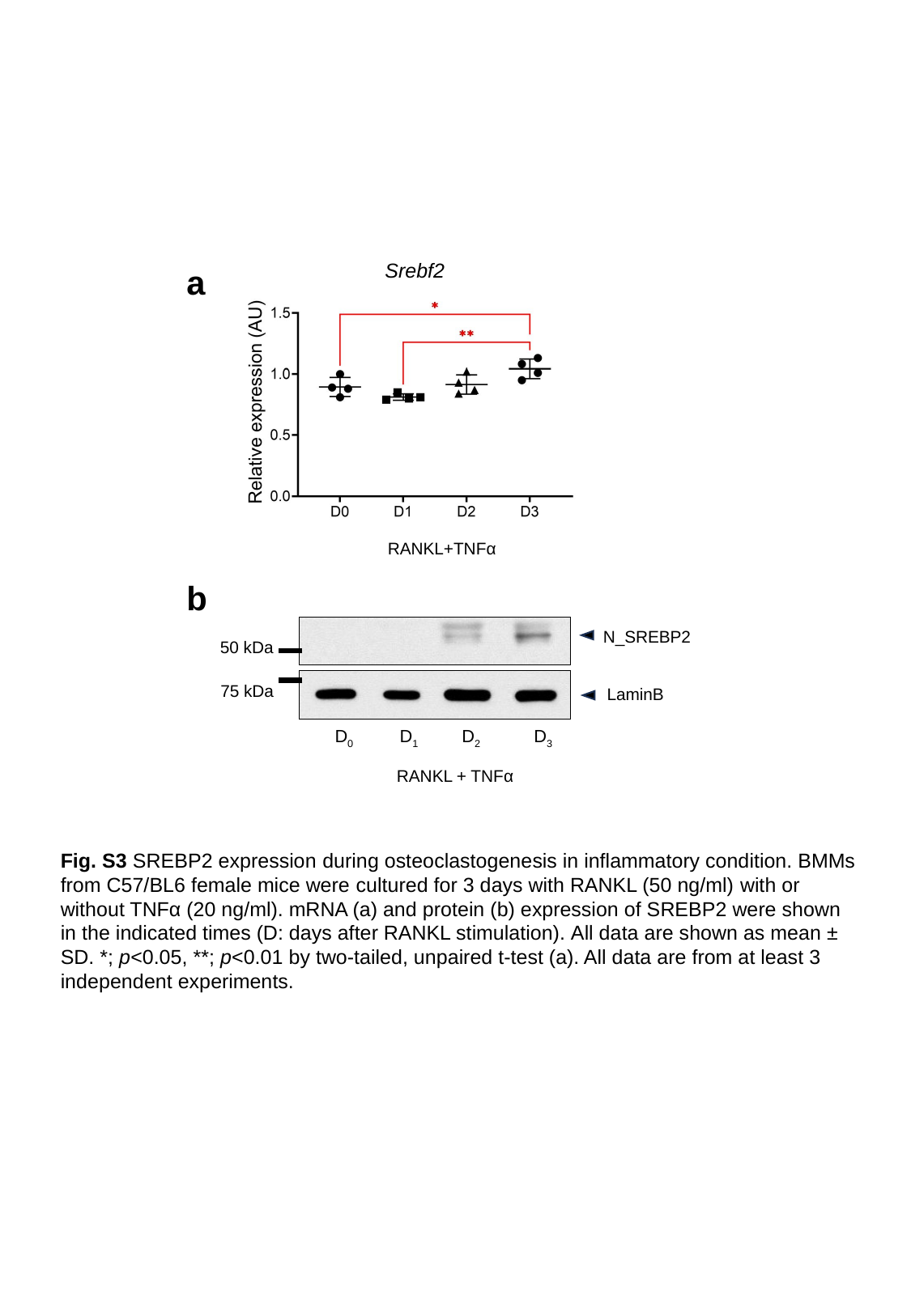

Srebf2
a
RANKL+TNFα
b
N_SREBP2
50 kDa
75 kDa
LaminB
D0 D1 D2 D3
RANKL + TNFα
Fig. S3 SREBP2 expression during osteoclastogenesis in inflammatory condition. BMMs from C57/BL6 female mice were cultured for 3 days with RANKL (50 ng/ml) with or without TNFα (20 ng/ml). mRNA (a) and protein (b) expression of SREBP2 were shown in the indicated times (D: days after RANKL stimulation). All data are shown as mean ± SD. *; p<0.05, **; p<0.01 by two-tailed, unpaired t-test (a). All data are from at least 3 independent experiments.

## Slide 5
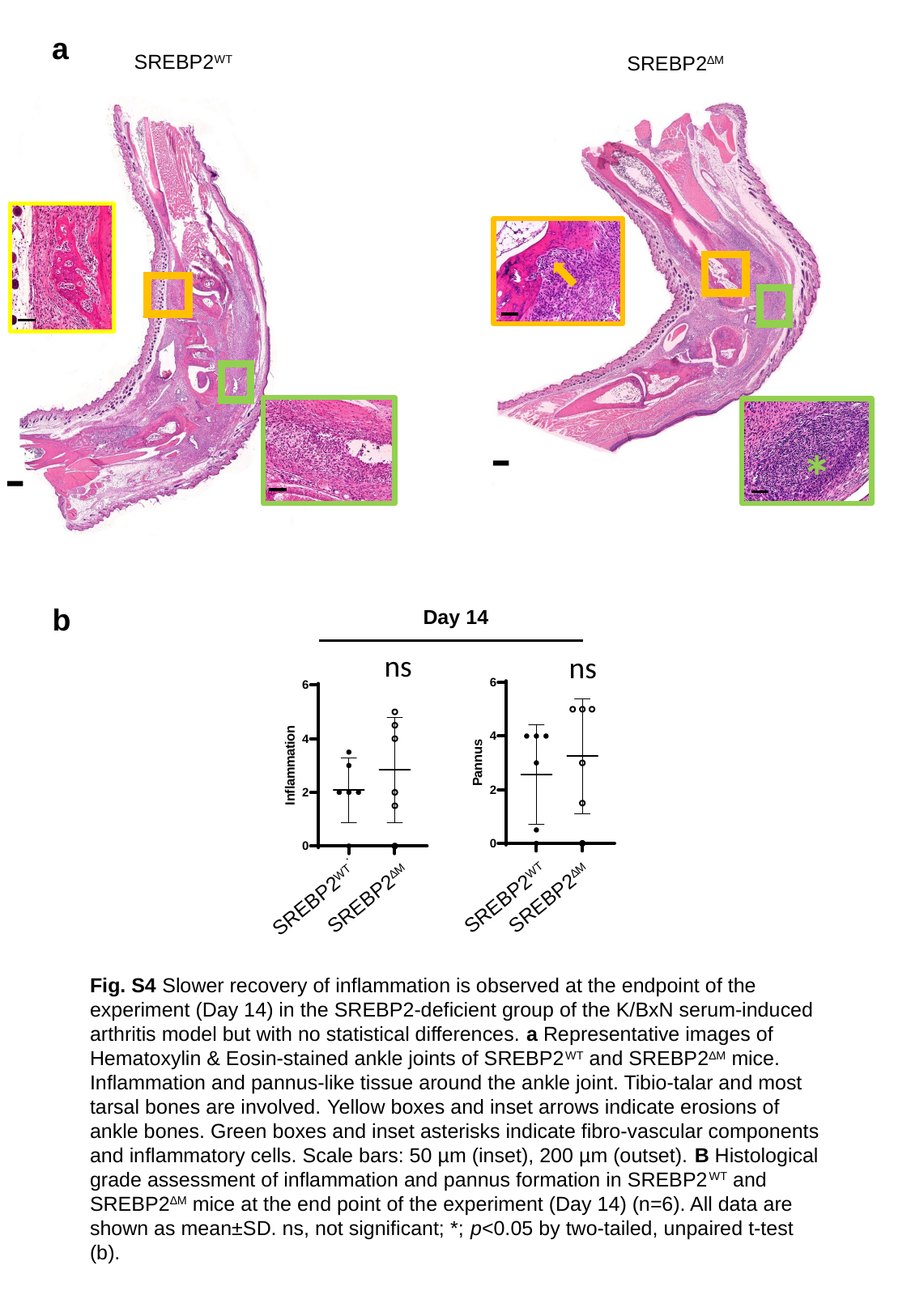

a
SREBP2WT
SREBP2∆M
*
b
Day 14
ns
ns
SREBP2WT
SREBP2∆M
SREBP2∆M
SREBP2WT
Fig. S4 Slower recovery of inflammation is observed at the endpoint of the experiment (Day 14) in the SREBP2-deficient group of the K/BxN serum-induced arthritis model but with no statistical differences. a Representative images of Hematoxylin & Eosin-stained ankle joints of SREBP2WT and SREBP2∆M mice. Inflammation and pannus-like tissue around the ankle joint. Tibio-talar and most tarsal bones are involved. Yellow boxes and inset arrows indicate erosions of ankle bones. Green boxes and inset asterisks indicate fibro-vascular components and inflammatory cells. Scale bars: 50 µm (inset), 200 µm (outset). B Histological grade assessment of inflammation and pannus formation in SREBP2WT and SREBP2∆M mice at the end point of the experiment (Day 14) (n=6). All data are shown as mean±SD. ns, not significant; *; p<0.05 by two-tailed, unpaired t-test (b).

## Slide 6
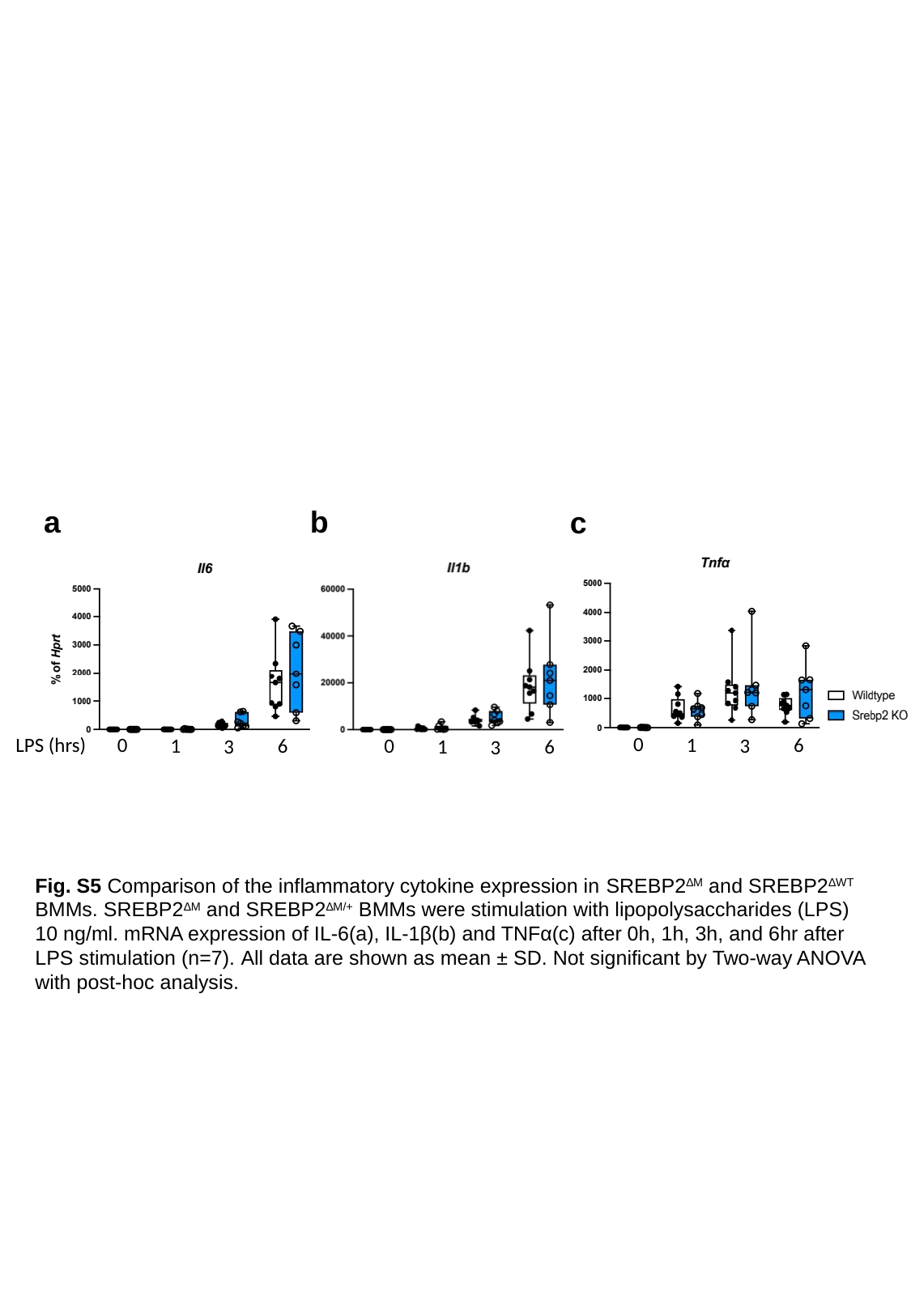

a
b
c
0
1
6
3
LPS (hrs)
0
1
6
0
1
6
3
3
Fig. S5 Comparison of the inflammatory cytokine expression in SREBP2∆M and SREBP2∆WT BMMs. SREBP2∆M and SREBP2∆M/+ BMMs were stimulation with lipopolysaccharides (LPS) 10 ng/ml. mRNA expression of IL-6(a), IL-1β(b) and TNFα(c) after 0h, 1h, 3h, and 6hr after LPS stimulation (n=7). All data are shown as mean ± SD. Not significant by Two-way ANOVA with post-hoc analysis.

## Slide 7
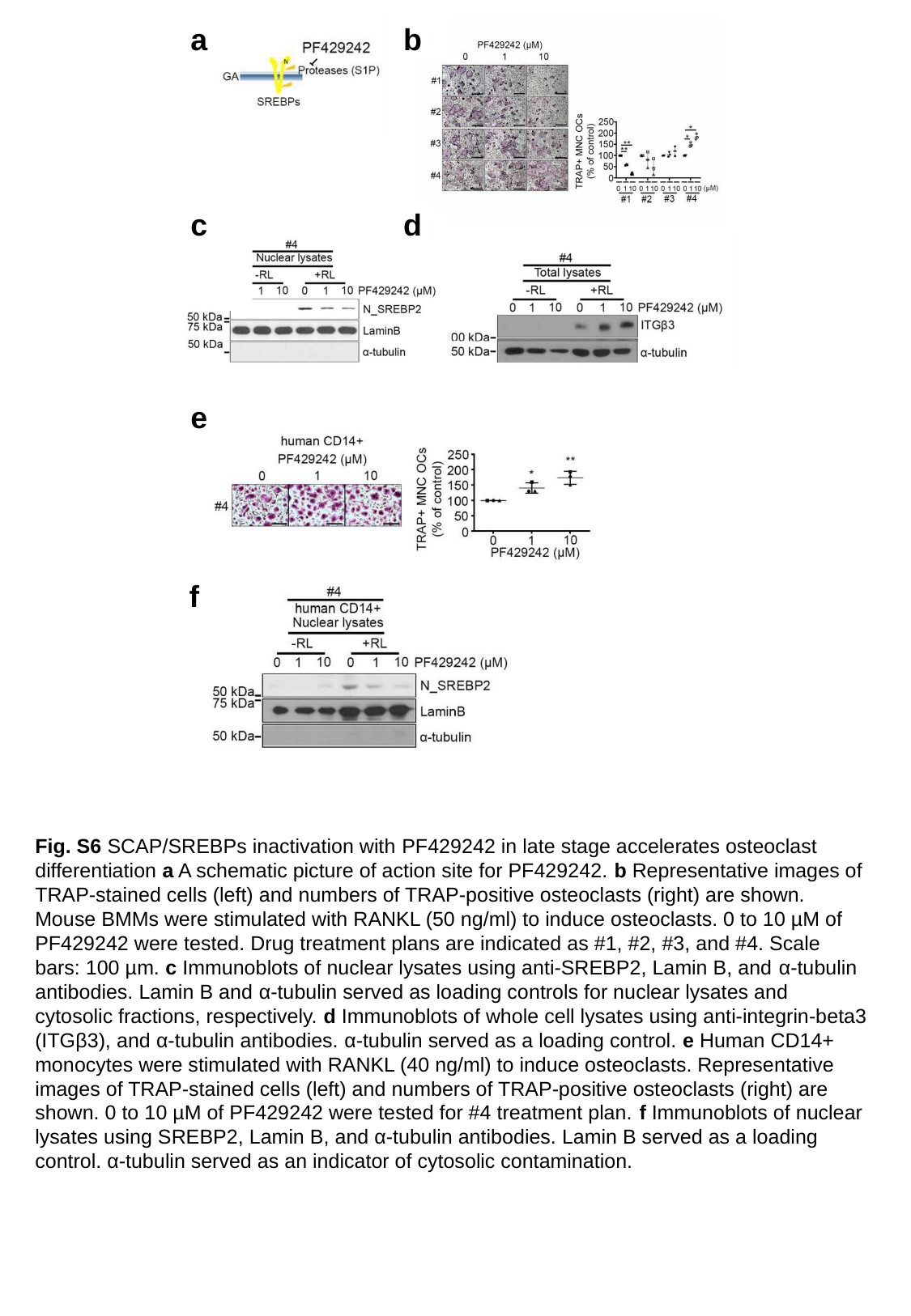

b
a
c
d
e
F
f
Fig. S6 SCAP/SREBPs inactivation with PF429242 in late stage accelerates osteoclast differentiation a A schematic picture of action site for PF429242. b Representative images of TRAP-stained cells (left) and numbers of TRAP-positive osteoclasts (right) are shown. Mouse BMMs were stimulated with RANKL (50 ng/ml) to induce osteoclasts. 0 to 10 µM of PF429242 were tested. Drug treatment plans are indicated as #1, #2, #3, and #4. Scale bars: 100 µm. c Immunoblots of nuclear lysates using anti-SREBP2, Lamin B, and α-tubulin antibodies. Lamin B and α-tubulin served as loading controls for nuclear lysates and cytosolic fractions, respectively. d Immunoblots of whole cell lysates using anti-integrin-beta3 (ITGβ3), and α-tubulin antibodies. α-tubulin served as a loading control. e Human CD14+ monocytes were stimulated with RANKL (40 ng/ml) to induce osteoclasts. Representative images of TRAP-stained cells (left) and numbers of TRAP-positive osteoclasts (right) are shown. 0 to 10 µM of PF429242 were tested for #4 treatment plan. f Immunoblots of nuclear lysates using SREBP2, Lamin B, and α-tubulin antibodies. Lamin B served as a loading control. α-tubulin served as an indicator of cytosolic contamination.

## Slide 8
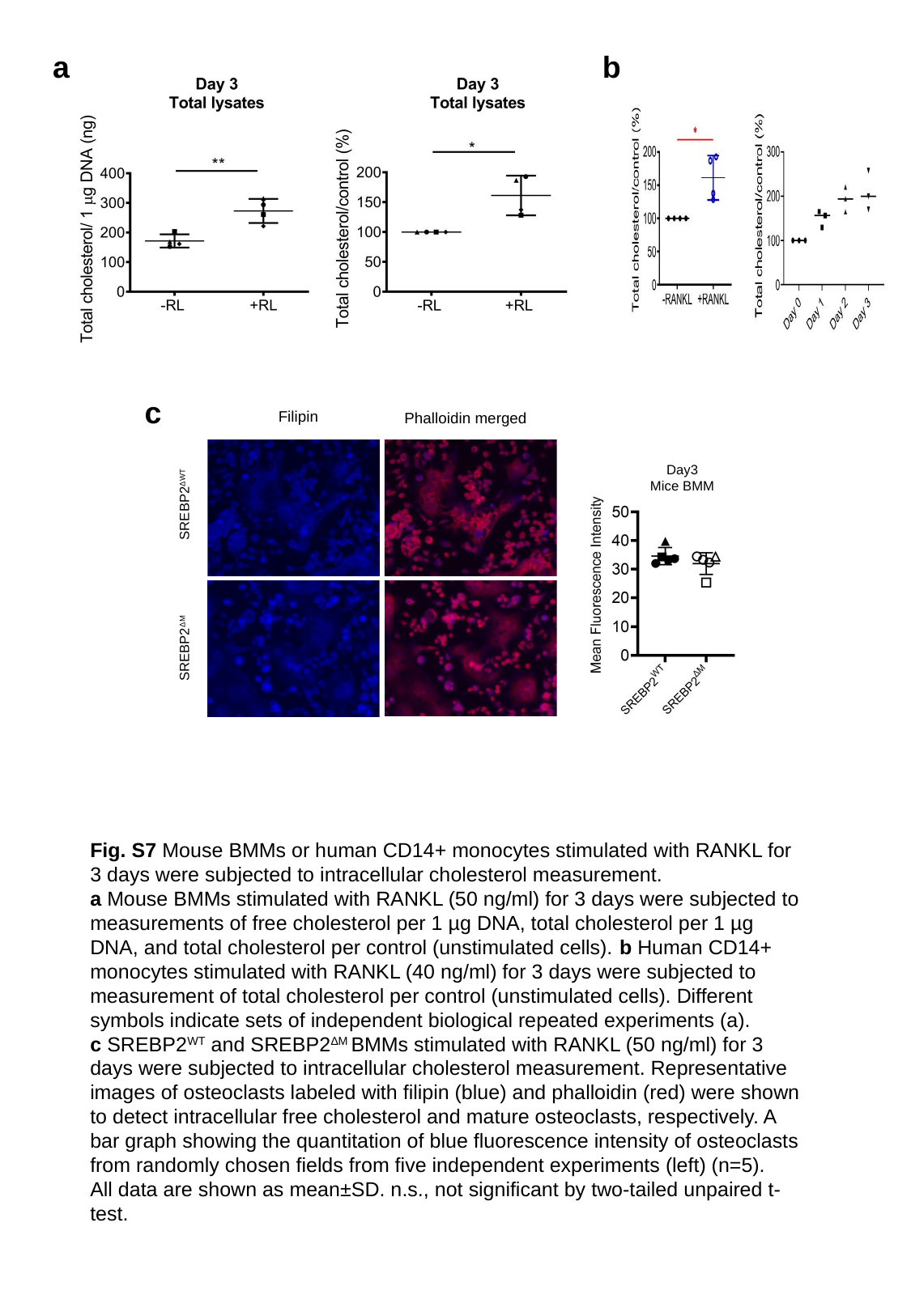

a
b
c
Filipin
Phalloidin merged
Day3
Mice BMM
SREBP2ΔWT
SREBP2ΔM
Fig. S7 Mouse BMMs or human CD14+ monocytes stimulated with RANKL for 3 days were subjected to intracellular cholesterol measurement.
a Mouse BMMs stimulated with RANKL (50 ng/ml) for 3 days were subjected to measurements of free cholesterol per 1 µg DNA, total cholesterol per 1 µg DNA, and total cholesterol per control (unstimulated cells). b Human CD14+ monocytes stimulated with RANKL (40 ng/ml) for 3 days were subjected to measurement of total cholesterol per control (unstimulated cells). Different symbols indicate sets of independent biological repeated experiments (a).
c SREBP2WT and SREBP2∆M BMMs stimulated with RANKL (50 ng/ml) for 3 days were subjected to intracellular cholesterol measurement. Representative images of osteoclasts labeled with filipin (blue) and phalloidin (red) were shown to detect intracellular free cholesterol and mature osteoclasts, respectively. A bar graph showing the quantitation of blue fluorescence intensity of osteoclasts from randomly chosen fields from five independent experiments (left) (n=5).
All data are shown as mean±SD. n.s., not significant by two-tailed unpaired t-test.

## Slide 9
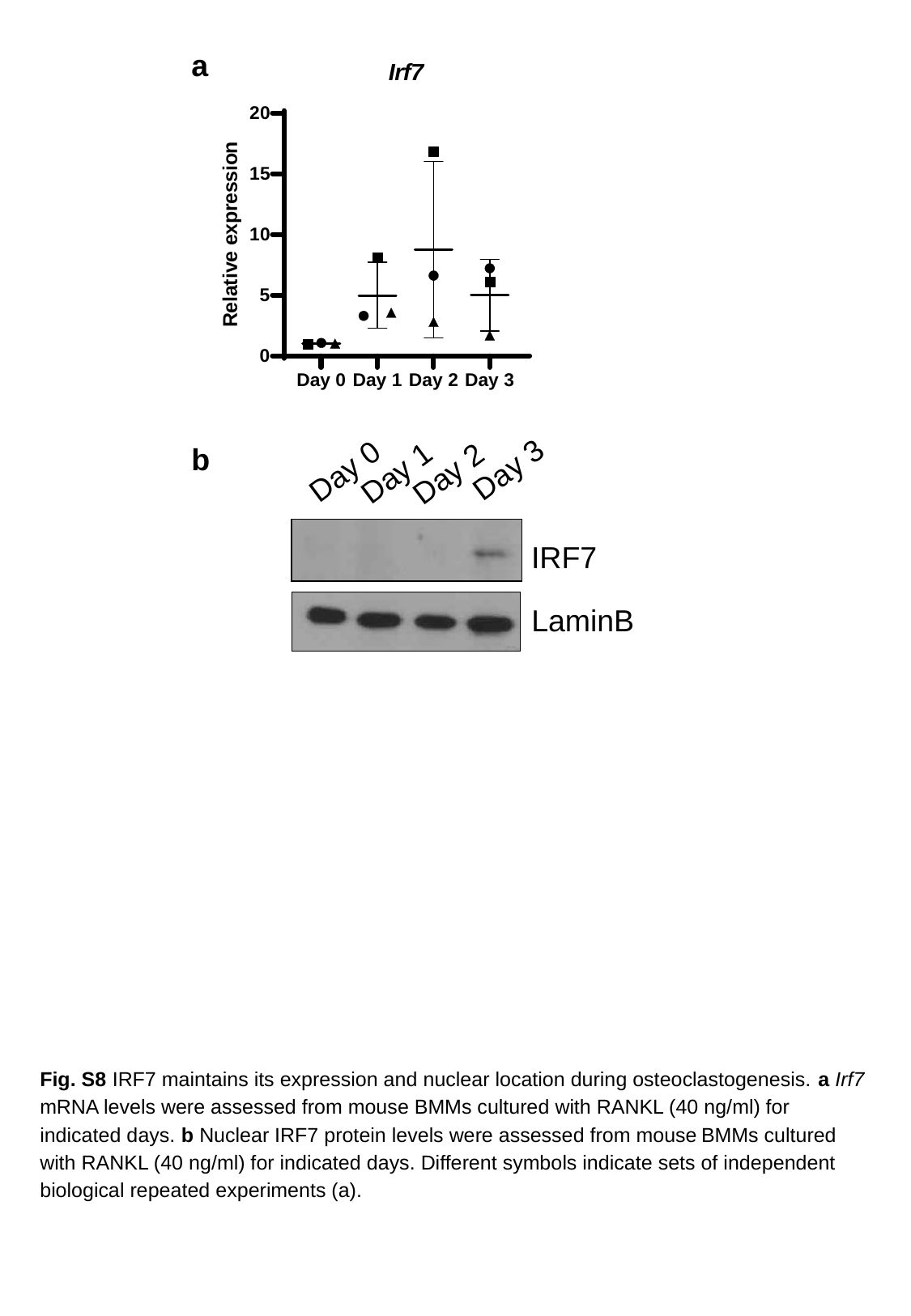

a
b
Day 3
Day 0
Day 1
Day 2
IRF7
LaminB
Fig. S8 IRF7 maintains its expression and nuclear location during osteoclastogenesis. a Irf7 mRNA levels were assessed from mouse BMMs cultured with RANKL (40 ng/ml) for indicated days. b Nuclear IRF7 protein levels were assessed from mouse BMMs cultured with RANKL (40 ng/ml) for indicated days. Different symbols indicate sets of independent biological repeated experiments (a).

## Slide 10
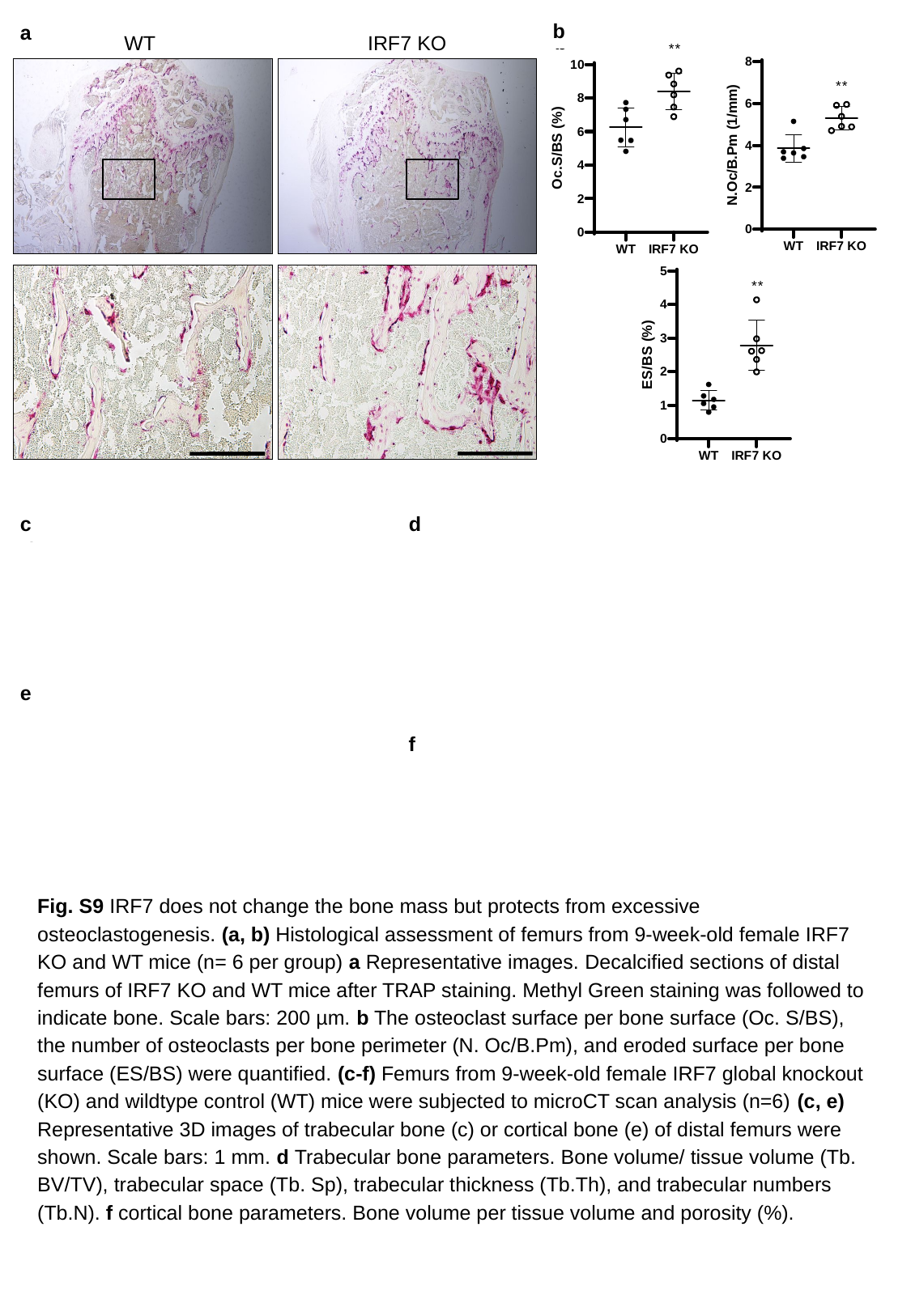

b
a
b
WT
IRF7 KO
d
c
d
c
e
e
f
f
Fig. S9 IRF7 does not change the bone mass but protects from excessive osteoclastogenesis. (a, b) Histological assessment of femurs from 9-week-old female IRF7 KO and WT mice (n= 6 per group) a Representative images. Decalcified sections of distal femurs of IRF7 KO and WT mice after TRAP staining. Methyl Green staining was followed to indicate bone. Scale bars: 200 µm. b The osteoclast surface per bone surface (Oc. S/BS), the number of osteoclasts per bone perimeter (N. Oc/B.Pm), and eroded surface per bone surface (ES/BS) were quantified. (c-f) Femurs from 9-week-old female IRF7 global knockout (KO) and wildtype control (WT) mice were subjected to microCT scan analysis (n=6) (c, e) Representative 3D images of trabecular bone (c) or cortical bone (e) of distal femurs were shown. Scale bars: 1 mm. d Trabecular bone parameters. Bone volume/ tissue volume (Tb. BV/TV), trabecular space (Tb. Sp), trabecular thickness (Tb.Th), and trabecular numbers (Tb.N). f cortical bone parameters. Bone volume per tissue volume and porosity (%).

## Slide 11
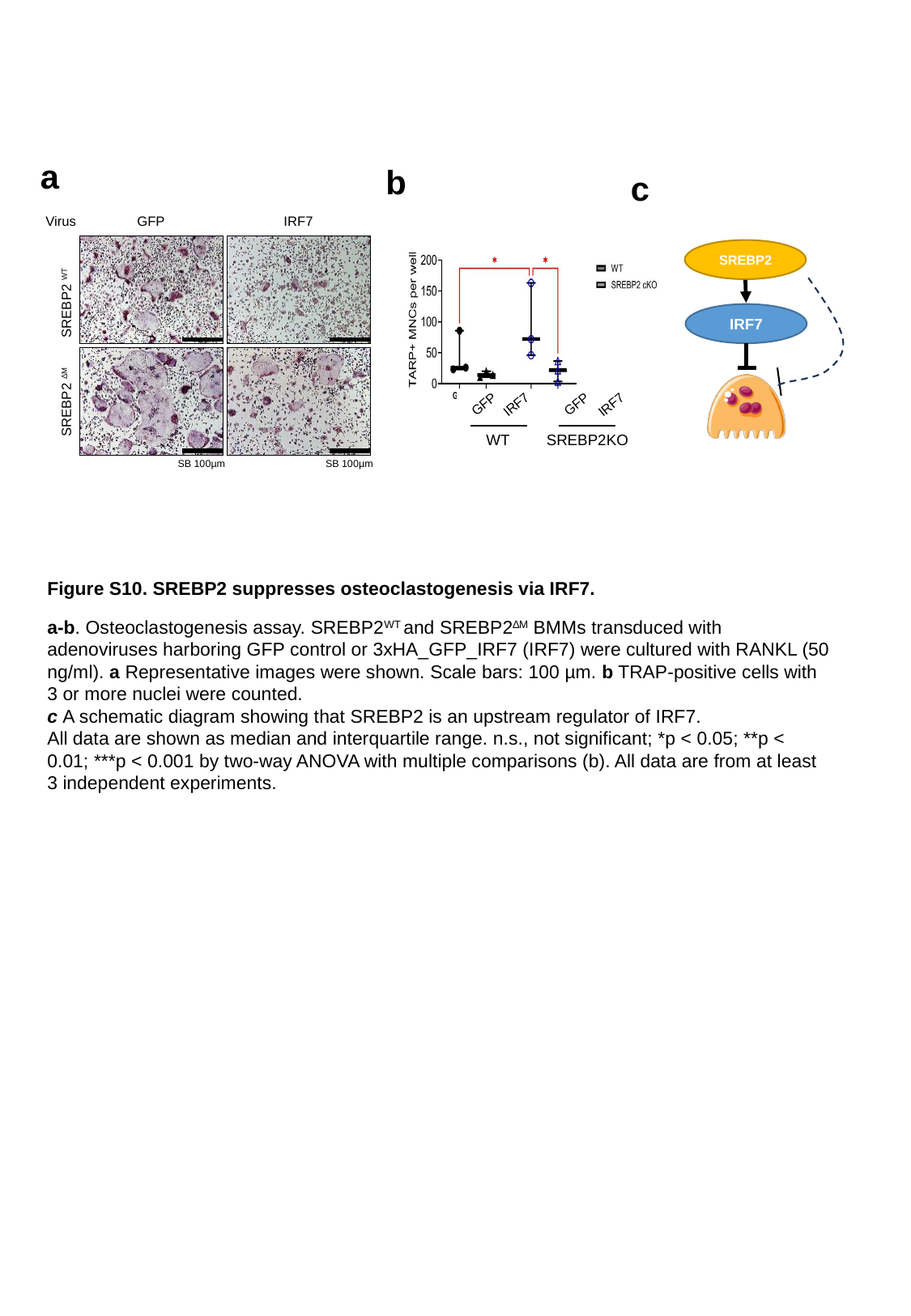

a
b
c
Virus
GFP
IRF7
SREBP2 WT
SREBP2 ΔM
SREBP2
IRF7
GFP
IRF7
IRF7
GFP
WT
SREBP2KO
SB 100µm
SB 100µm
Figure S10. SREBP2 suppresses osteoclastogenesis via IRF7.
a-b. Osteoclastogenesis assay. SREBP2WT and SREBP2∆M BMMs transduced with adenoviruses harboring GFP control or 3xHA_GFP_IRF7 (IRF7) were cultured with RANKL (50 ng/ml). a Representative images were shown. Scale bars: 100 µm. b TRAP-positive cells with 3 or more nuclei were counted.
c A schematic diagram showing that SREBP2 is an upstream regulator of IRF7.
All data are shown as median and interquartile range. n.s., not significant; *p < 0.05; **p < 0.01; ***p < 0.001 by two-way ANOVA with multiple comparisons (b). All data are from at least 3 independent experiments.

## Slide 12
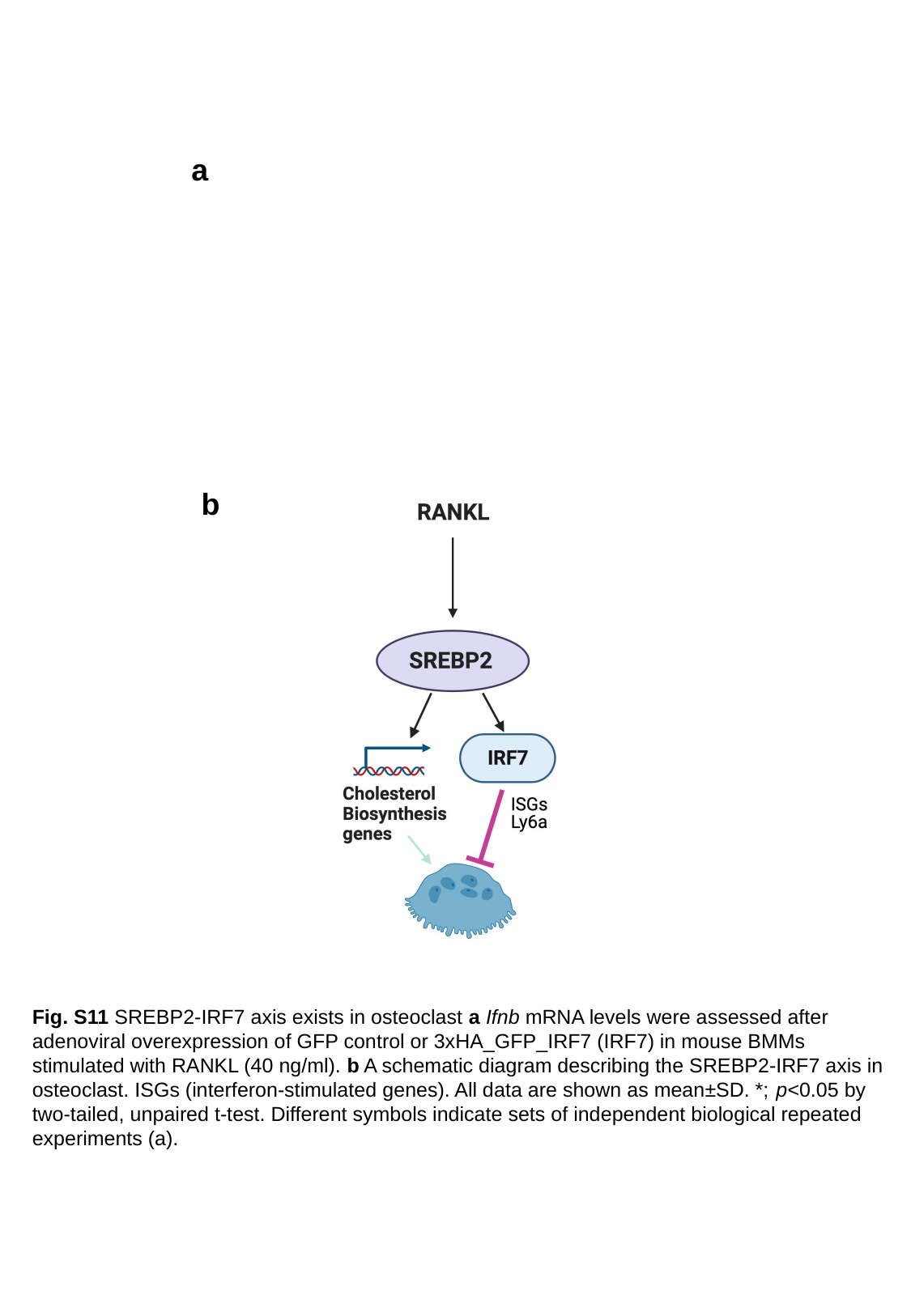

a
b
Fig. S11 SREBP2-IRF7 axis exists in osteoclast a Ifnb mRNA levels were assessed after adenoviral overexpression of GFP control or 3xHA_GFP_IRF7 (IRF7) in mouse BMMs stimulated with RANKL (40 ng/ml). b A schematic diagram describing the SREBP2-IRF7 axis in osteoclast. ISGs (interferon-stimulated genes). All data are shown as mean±SD. *; p<0.05 by two-tailed, unpaired t-test. Different symbols indicate sets of independent biological repeated experiments (a).

## Slide 13
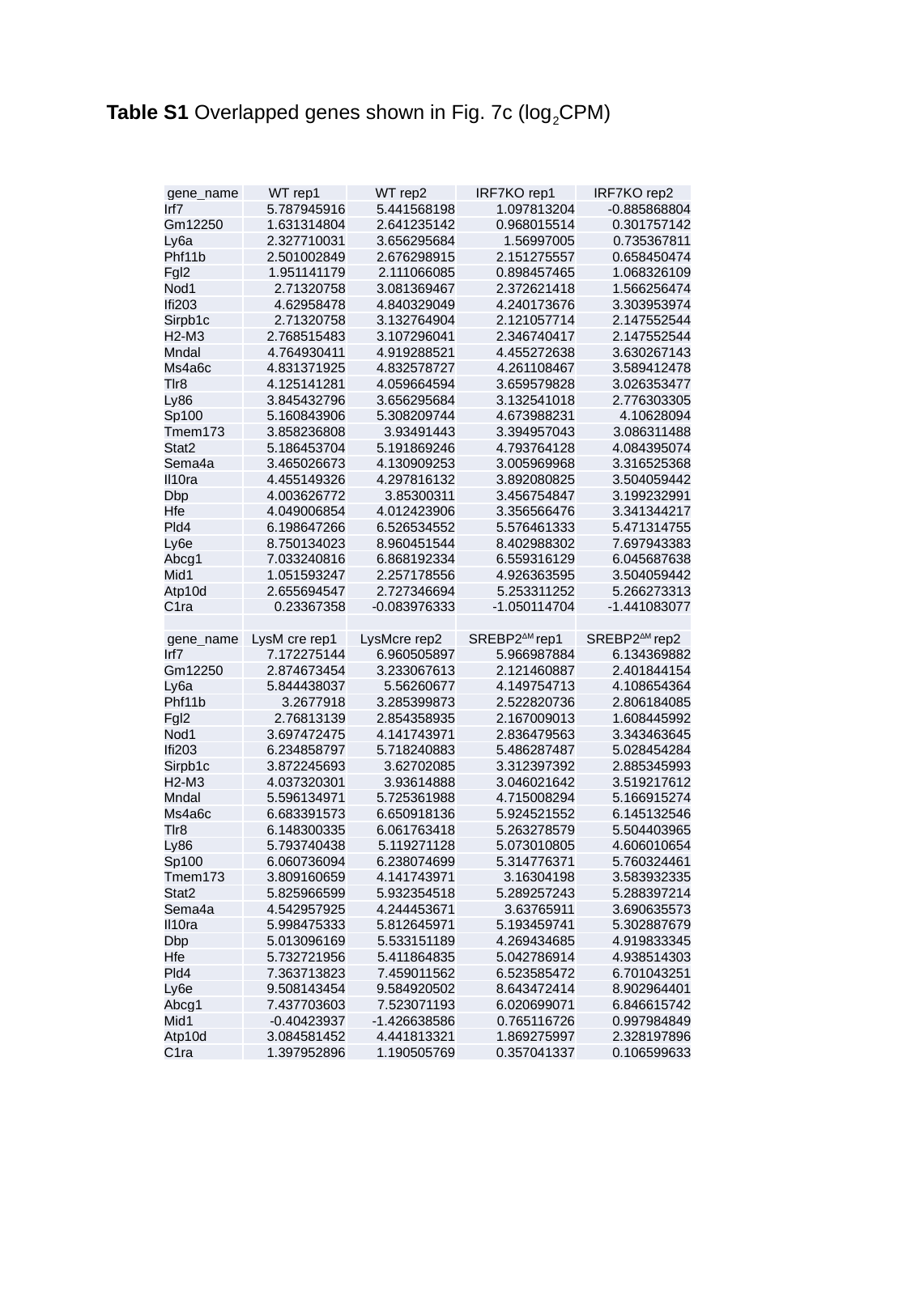

Table S1 Overlapped genes shown in Fig. 7c (log2CPM)
| gene\_name | WT rep1 | WT rep2 | IRF7KO rep1 | IRF7KO rep2 |
| --- | --- | --- | --- | --- |
| Irf7 | 5.787945916 | 5.441568198 | 1.097813204 | -0.885868804 |
| Gm12250 | 1.631314804 | 2.641235142 | 0.968015514 | 0.301757142 |
| Ly6a | 2.327710031 | 3.656295684 | 1.56997005 | 0.735367811 |
| Phf11b | 2.501002849 | 2.676298915 | 2.151275557 | 0.658450474 |
| Fgl2 | 1.951141179 | 2.111066085 | 0.898457465 | 1.068326109 |
| Nod1 | 2.71320758 | 3.081369467 | 2.372621418 | 1.566256474 |
| Ifi203 | 4.62958478 | 4.840329049 | 4.240173676 | 3.303953974 |
| Sirpb1c | 2.71320758 | 3.132764904 | 2.121057714 | 2.147552544 |
| H2-M3 | 2.768515483 | 3.107296041 | 2.346740417 | 2.147552544 |
| Mndal | 4.764930411 | 4.919288521 | 4.455272638 | 3.630267143 |
| Ms4a6c | 4.831371925 | 4.832578727 | 4.261108467 | 3.589412478 |
| Tlr8 | 4.125141281 | 4.059664594 | 3.659579828 | 3.026353477 |
| Ly86 | 3.845432796 | 3.656295684 | 3.132541018 | 2.776303305 |
| Sp100 | 5.160843906 | 5.308209744 | 4.673988231 | 4.10628094 |
| Tmem173 | 3.858236808 | 3.93491443 | 3.394957043 | 3.086311488 |
| Stat2 | 5.186453704 | 5.191869246 | 4.793764128 | 4.084395074 |
| Sema4a | 3.465026673 | 4.130909253 | 3.005969968 | 3.316525368 |
| Il10ra | 4.455149326 | 4.297816132 | 3.892080825 | 3.504059442 |
| Dbp | 4.003626772 | 3.85300311 | 3.456754847 | 3.199232991 |
| Hfe | 4.049006854 | 4.012423906 | 3.356566476 | 3.341344217 |
| Pld4 | 6.198647266 | 6.526534552 | 5.576461333 | 5.471314755 |
| Ly6e | 8.750134023 | 8.960451544 | 8.402988302 | 7.697943383 |
| Abcg1 | 7.033240816 | 6.868192334 | 6.559316129 | 6.045687638 |
| Mid1 | 1.051593247 | 2.257178556 | 4.926363595 | 3.504059442 |
| Atp10d | 2.655694547 | 2.727346694 | 5.253311252 | 5.266273313 |
| C1ra | 0.23367358 | -0.083976333 | -1.050114704 | -1.441083077 |
| | | | | |
| gene\_name | LysM cre rep1 | LysMcre rep2 | SREBP2∆M rep1 | SREBP2∆M rep2 |
| Irf7 | 7.172275144 | 6.960505897 | 5.966987884 | 6.134369882 |
| Gm12250 | 2.874673454 | 3.233067613 | 2.121460887 | 2.401844154 |
| Ly6a | 5.844438037 | 5.56260677 | 4.149754713 | 4.108654364 |
| Phf11b | 3.2677918 | 3.285399873 | 2.522820736 | 2.806184085 |
| Fgl2 | 2.76813139 | 2.854358935 | 2.167009013 | 1.608445992 |
| Nod1 | 3.697472475 | 4.141743971 | 2.836479563 | 3.343463645 |
| Ifi203 | 6.234858797 | 5.718240883 | 5.486287487 | 5.028454284 |
| Sirpb1c | 3.872245693 | 3.62702085 | 3.312397392 | 2.885345993 |
| H2-M3 | 4.037320301 | 3.93614888 | 3.046021642 | 3.519217612 |
| Mndal | 5.596134971 | 5.725361988 | 4.715008294 | 5.166915274 |
| Ms4a6c | 6.683391573 | 6.650918136 | 5.924521552 | 6.145132546 |
| Tlr8 | 6.148300335 | 6.061763418 | 5.263278579 | 5.504403965 |
| Ly86 | 5.793740438 | 5.119271128 | 5.073010805 | 4.606010654 |
| Sp100 | 6.060736094 | 6.238074699 | 5.314776371 | 5.760324461 |
| Tmem173 | 3.809160659 | 4.141743971 | 3.16304198 | 3.583932335 |
| Stat2 | 5.825966599 | 5.932354518 | 5.289257243 | 5.288397214 |
| Sema4a | 4.542957925 | 4.244453671 | 3.63765911 | 3.690635573 |
| Il10ra | 5.998475333 | 5.812645971 | 5.193459741 | 5.302887679 |
| Dbp | 5.013096169 | 5.533151189 | 4.269434685 | 4.919833345 |
| Hfe | 5.732721956 | 5.411864835 | 5.042786914 | 4.938514303 |
| Pld4 | 7.363713823 | 7.459011562 | 6.523585472 | 6.701043251 |
| Ly6e | 9.508143454 | 9.584920502 | 8.643472414 | 8.902964401 |
| Abcg1 | 7.437703603 | 7.523071193 | 6.020699071 | 6.846615742 |
| Mid1 | -0.40423937 | -1.426638586 | 0.765116726 | 0.997984849 |
| Atp10d | 3.084581452 | 4.441813321 | 1.869275997 | 2.328197896 |
| C1ra | 1.397952896 | 1.190505769 | 0.357041337 | 0.106599633 |

## Slide 14
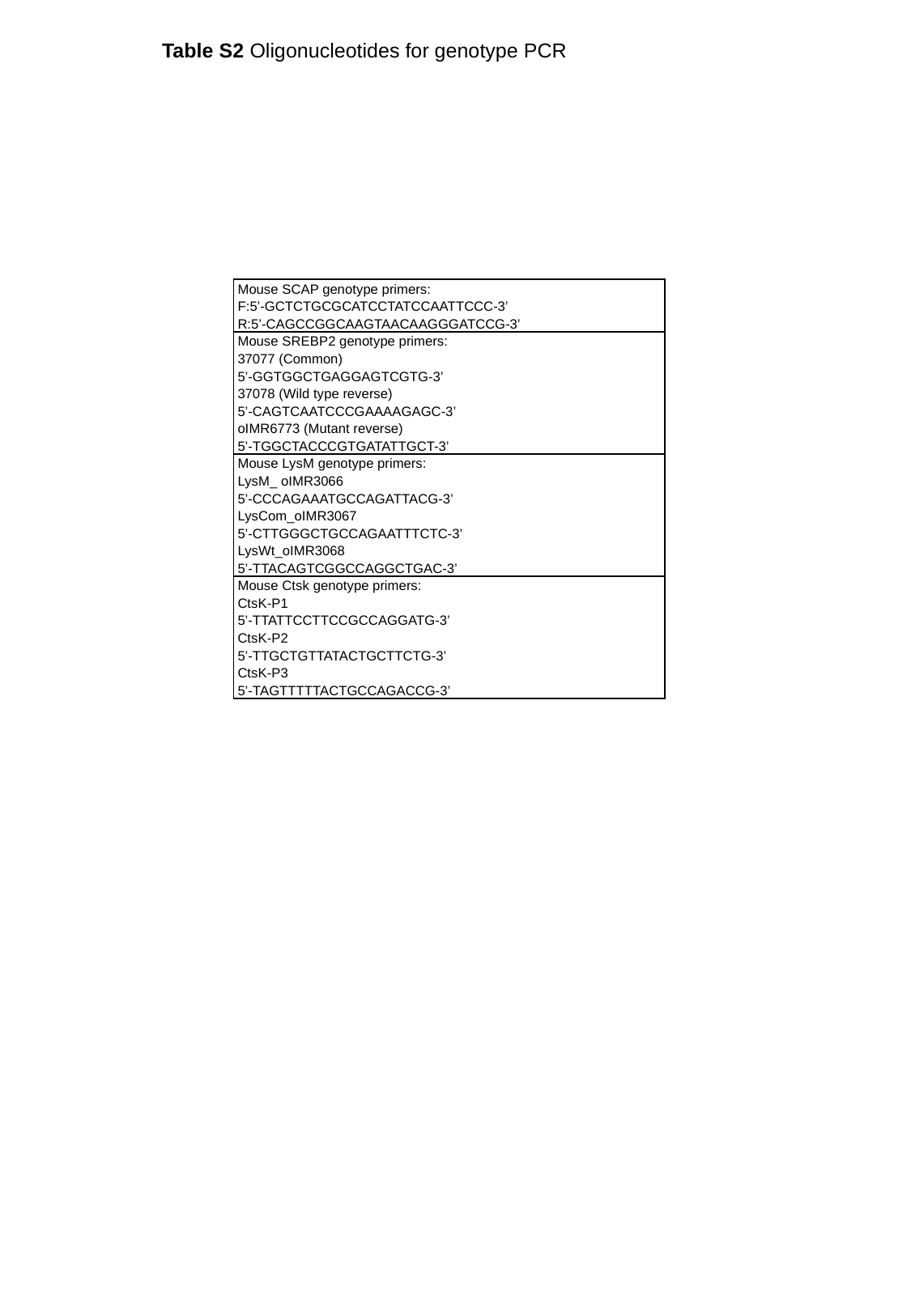

Table S2 Oligonucleotides for genotype PCR
| Mouse SCAP genotype primers: F:5’-GCTCTGCGCATCCTATCCAATTCCC-3’ R:5’-CAGCCGGCAAGTAACAAGGGATCCG-3’ |
| --- |
| Mouse SREBP2 genotype primers: 37077 (Common) 5’-GGTGGCTGAGGAGTCGTG-3’ 37078 (Wild type reverse) 5’-CAGTCAATCCCGAAAAGAGC-3’ oIMR6773 (Mutant reverse) 5’-TGGCTACCCGTGATATTGCT-3’ |
| Mouse LysM genotype primers: LysM\_ oIMR3066 5’-CCCAGAAATGCCAGATTACG-3’ LysCom\_oIMR3067 5’-CTTGGGCTGCCAGAATTTCTC-3’ LysWt\_oIMR3068 5’-TTACAGTCGGCCAGGCTGAC-3’ |
| Mouse Ctsk genotype primers: CtsK-P1 5’-TTATTCCTTCCGCCAGGATG-3’ CtsK-P2 5’-TTGCTGTTATACTGCTTCTG-3’ CtsK-P3 5’-TAGTTTTTACTGCCAGACCG-3’ |

## Slide 15
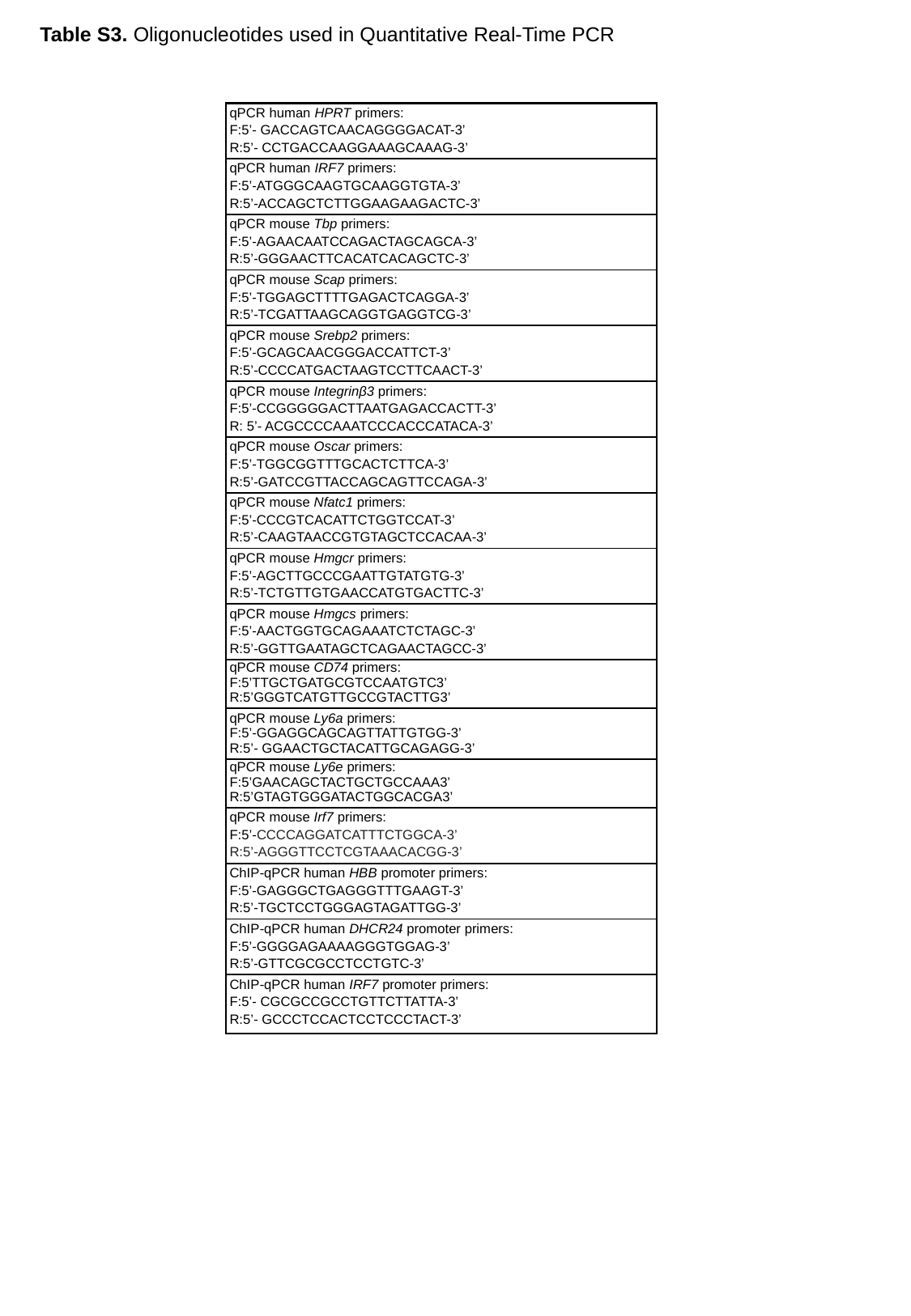

Table S3. Oligonucleotides used in Quantitative Real-Time PCR
| qPCR human HPRT primers: F:5’- GACCAGTCAACAGGGGACAT-3’ R:5’- CCTGACCAAGGAAAGCAAAG-3’ |
| --- |
| qPCR human IRF7 primers: F:5’-ATGGGCAAGTGCAAGGTGTA-3’ R:5’-ACCAGCTCTTGGAAGAAGACTC-3’ |
| qPCR mouse Tbp primers: F:5’-AGAACAATCCAGACTAGCAGCA-3’ R:5’-GGGAACTTCACATCACAGCTC-3’ |
| qPCR mouse Scap primers: F:5’-TGGAGCTTTTGAGACTCAGGA-3’ R:5’-TCGATTAAGCAGGTGAGGTCG-3’ |
| qPCR mouse Srebp2 primers: F:5’-GCAGCAACGGGACCATTCT-3’ R:5’-CCCCATGACTAAGTCCTTCAACT-3’ |
| qPCR mouse Integrinβ3 primers: F:5’-CCGGGGGACTTAATGAGACCACTT-3’ R: 5’- ACGCCCCAAATCCCACCCATACA-3’ |
| qPCR mouse Oscar primers: F:5’-TGGCGGTTTGCACTCTTCA-3’ R:5’-GATCCGTTACCAGCAGTTCCAGA-3’ |
| qPCR mouse Nfatc1 primers: F:5’-CCCGTCACATTCTGGTCCAT-3’ R:5’-CAAGTAACCGTGTAGCTCCACAA-3’ |
| qPCR mouse Hmgcr primers: F:5’-AGCTTGCCCGAATTGTATGTG-3’ R:5’-TCTGTTGTGAACCATGTGACTTC-3’ |
| qPCR mouse Hmgcs primers: F:5’-AACTGGTGCAGAAATCTCTAGC-3’ R:5’-GGTTGAATAGCTCAGAACTAGCC-3’ |
| qPCR mouse CD74 primers: F:5’TTGCTGATGCGTCCAATGTC3’ R:5’GGGTCATGTTGCCGTACTTG3’ |
| qPCR mouse Ly6a primers: F:5’-GGAGGCAGCAGTTATTGTGG-3’ R:5’- GGAACTGCTACATTGCAGAGG-3’ |
| qPCR mouse Ly6e primers: F:5’GAACAGCTACTGCTGCCAAA3’ R:5’GTAGTGGGATACTGGCACGA3’ |
| qPCR mouse Irf7 primers: F:5’-CCCCAGGATCATTTCTGGCA-3’ R:5’-AGGGTTCCTCGTAAACACGG-3’ |
| ChIP-qPCR human HBB promoter primers: F:5’-GAGGGCTGAGGGTTTGAAGT-3’ R:5’-TGCTCCTGGGAGTAGATTGG-3’ |
| ChIP-qPCR human DHCR24 promoter primers: F:5’-GGGGAGAAAAGGGTGGAG-3’ R:5’-GTTCGCGCCTCCTGTC-3’ |
| ChIP-qPCR human IRF7 promoter primers: F:5’- CGCGCCGCCTGTTCTTATTA-3’ R:5’- GCCCTCCACTCCTCCCTACT-3’ |
